# Substrate‐Enabled Room‐Temperature Electrochemical Deposition of Crystalline ZnMnO_3_

**DOI:** 10.1002/cphc.202200586

**Published:** 2022-10-25

**Authors:** Karin Rettenmaier, Gregor A. Zickler, Günther J. Redhammer, Thomas Berger

**Affiliations:** ^1^ Department of Chemistry and Physics of Materials University of Salzburg Jakob-Haringer-Straße 2a A-5020 Salzburg Austria

**Keywords:** electrodeposition, crystal growth, ternary metal oxides, nanostructures, electrodes

## Abstract

Mixed transition metal oxides have emerged as promising electrode materials for electrochemical energy storage and conversion. To optimize the functional electrode properties, synthesis approaches allowing for a systematic tailoring of the materials’ composition, crystal structure and morphology are urgently needed. Here we report on the room‐temperature electrodeposition of a ternary oxide based on earth‐abundant metals, specifically, the defective cubic spinel ZnMnO_3_. In this unprecedented approach, ZnO surfaces act as (i) electron source for the interfacial reduction of MnO_4_^−^ in aqueous solution, (ii) as substrate for epitaxial growth of the deposit and (iii) as Zn precursor for the formation of ZnMnO_3_. Epitaxial growth of ZnMnO_3_ on the lateral facets of ZnO nanowires assures effective electronic communication between the electroactive material and the conducting scaffold and gives rise to a pronounced 2‐dimensional morphology of the electrodeposit forming – after partial delamination from the substrate – twisted nanosheets. The synthesis strategy shows promise for the direct growth of different mixed transition metal oxides as electroactive phase onto conductive substrates and thus for the fabrication of binder‐free nanocomposite electrodes.

## Graphical abstract

**Lateral facets** of wurtzite ZnO nanowires serve as precursor and substrate for the epitaxial growth of defective cubic spinel ZnMnO_3_ via a room‐temperature, one‐step electrodeposition process from aqueous KMnO_4_ solution.

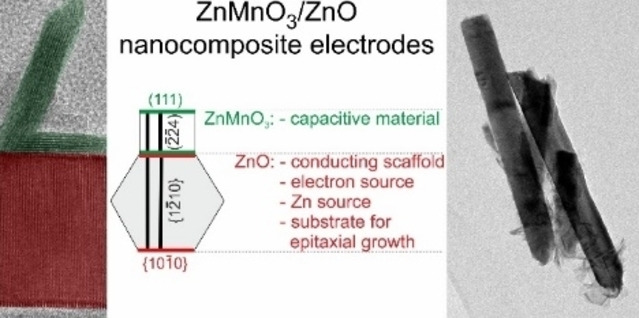

## Introduction

The search for energy storage technologies combining high performance with economic and environmental sustainability has triggered extensive research on manganese oxides as active materials in ion batteries and supercapacitors.[^1^, ^2^, ^3^, ^4^] Importantly, related research endeavors are motivated by the prospect of using manganese oxide electrodes in charge storage devices based on aqueous electrolytes. Furthermore, various environmentally benign synthesis routes are available nowadays.^[4]^ However, binary oxides often suffer from some shortcomings such as inherently low ionic and/or electronic conductivities, which hamper their broad application.[^1^, ^2^, ^3^] A way to compensate for low ionic conductivities in electroactive materials consists in the synthesis of morphologically and structurally well‐defined nanoobjects, which combine short solid‐state diffusion distances for ions with structural features allowing for fast ion transport.[^1^, ^3^] However, when assembling such nanoobjects into electrodes great care must be taken to assure high contact areas between the electroactive material and the electrolyte.^[3]^ This is especially challenging when the material is synthesized in the form of a powder, which then has to be processed together with binders and conductive additives into slurries, followed by electrode fabrication e. g. by casting methods. In contrast, direct growth of electroactive nanostructures onto porous and conductive substrates allows for the fabrication of binder‐free electrodes. The resulting composite structures sometimes alleviate the problem of low electronic conductivities of the electroactive material provided that the diffusion path lengths for electrons are kept short.[^1^, ^3^] In addition, strategies aim at enhancing the intrinsic electronic conductivity of the electroactive material by manipulating the defect chemistry of the material e. g. by the incorporation of heteroatoms or the intentional creation of ion vacancies.[^1^, ^3^]

Mixed metal oxides (i. e. single‐phase ternary or multinary metal oxides) have recently emerged as promising electrode materials for ion batteries, metal‐air batteries and supercapacitors.[^5^, ^6^, ^7^, ^8^, ^9^] Importantly, mixed transition metal oxides typically exhibit higher electrical conductivities than binary oxides. This is due to the relatively low activation energies for electron transfer between cations featuring mixed valence states. Transition metal oxides in the spinel structure constitute particularly promising energy storage materials due to the fact that a wide range of cations in different oxidation states can be accommodated at the tetrahedral and octahedral sites of the oxygen sublattice. Spinel‐type ternary transition metal oxides denoted as A_x_B_3‐x_O_4_ (with A, B=Co, Zn, Ni, Fe, Cu, Mn…) may have a stoichiometric or a non‐stoichiometric composition and contain anion and/or cation vacancies. Importantly, physical and chemical properties of the material are determined by composition and site occupancy and their deliberate variation would in principle allow for a systematic preparation of materials with tunable properties.

One of the most extensively investigated ternary transition metal oxide spinel is NiCo_2_O_4_, which shows outstanding pseudocapacitive behavior.^[10]^ Efforts have been made to substitute cobalt by more earth‐abundant and eco‐friendly elements like manganese. Different manganates have been used as electrocatalysts[^11^, ^12^] and as electrode material in ion batteries^[5]^ including multivalent water‐based batteries.^[13]^ In the case of zinc manganese oxide, two different spinel types have been reported – tetragonal ZnMn_2_O_4_ and cubic ZnMnO_3_.^[14]^ ZnMn_2_O_4_ has been synthesized in different morphologies in the nanometer size regime and has been extensively investigated as electrode material in Li‐ion batteries.[^15^, ^16^] However, synthesis routes for phase pure ZnMnO_3_ nanomaterials became available only recently.[^14^, ^17^] This is why there exist only few reports on the use of ZnMnO_3_ as electroactive material so far. These studies have highlighted, however, a great potential of the material for applications in Li‐ion batteries[^18^, ^19^, ^20^, ^21^, ^22^, ^23^] and magnesium rechargeable batteries.^[24]^ The difficulty to synthesize ZnMnO_3_ in a phase pure form was attributed to the very narrow stabilization region of this defective cubic spinel.^[14]^ Nevertheless, various wet‐chemical synthesis methods yielding nanosized ZnMnO_3_ have been reported.[^14^, ^18^, ^19^, ^20^, ^21^, ^22^, ^23^, ^24^, ^25^, ^26^] These methods, however, require a thermal post‐synthesis treatment at temperatures between 300–650 °C to obtain the crystalline product. Remarkably, crystalline ZnMnO_3_ powders featuring a broad size distribution below 200 nm have been synthesized by a hydrothermal approach at a relatively low temperature of 180 °C.^[17]^

In general, the development of synthetic routes facilitating the minute control and variation of both structural and compositional material properties is challenging. However, only a proper tuning of crystal structure, morphology and composition together with minute defect engineering will allow to exploit the full potential of mixed transition metal oxides. For instance, it has been shown that spinel‐type mixed transition metal oxides consisting of two‐dimensional nanostructures may provide significantly improved properties when used in electrochemical energy storage applications.^[15]^ However, these intrinsically non‐layered materials can not be converted into two‐dimensional nanostructures via top‐down exfoliation methods.^[27]^ Consequently, there is an urgent need for simple bottom‐up strategies yielding mixed transition metal oxides featuring two‐dimensional morphologies.^[15]^

The task is further complicated when aiming at the preparation of binder‐free electrodes by the direct deposition of the electroactive material onto a high surface area conductive substrate. In this case, a deterioration of the scaffold properties must be avoided by the selection of proper deposition and processing conditions. At the same time, a good electronic communication between the active material and the substrate must be established. In addition, synthesis routes should ideally comply with the principles of modern green chemistry e. g. by relying on water‐based low temperature processes using non‐polluting reagents.

In this context, electrodeposition exhibits several advantages compared with other synthesis methods being a simple and low‐temperature one‐step process, which is well‐suited for large‐scale production.[^4^, ^6^] An efficient electron transfer between the conducting scaffold and the growing deposit is a prerequisite for efficient electrodeposition. Consequently, resulting composites typically ensure excellent electronic communication between the active material and the substrate, which is crucial for their successful application as electrode materials in energy storage devices. Recently, this method has been used, for instance, to deposit MnO_2_ nanostructures onto flexible substrates such as carbon‐fiber paper^[28]^ or graphene paper.^[29]^ The resulting composite electrodes exhibited outstanding performance in Zn/MnO_2_ batteries^[28]^ and supercapacitors.^[29]^

One‐step electrodeposition at low temperatures (*T*=80–200 °C) was even used to prepare some ternary transition metal oxides (such as Co_x_Fe_3‐x_O_4_, ZnFe_2_O_4_ and MgFe_2_O_4_) as dense polycrystalline films on Fe substrates^[30]^ or in the form of thin epitaxial films on Au substrates.[^31^, ^32^] In addition, polycrystalline dense films of alkaline‐earth tungstates and molybdates on W or Mo foils were deposited upon anodic dissolution of the substrate at room temperature.^[33]^

ZnO nanowire arrays feature electronic and chemical properties, which make them particularly interesting as substrates for electrodeposition.^[34]^ These materials have successfully been used as substrates for binary MnO_2_ ^[34]^ or as self‐sacrificial templates for ternary ZnMnO_3_,^[26]^ respectively. In these cases, however, additional processing steps such as high temperature annealing had to be performed to obtain the functional electrodes.

Here we report on the room‐temperature, one‐step electrodeposition of the defective cubic spinel ZnMnO_3_ onto ZnO nanowire arrays from an aqueous KMnO_4_ solution. The morphological and structural properties of the resulting nanocomposite films have been characterized by electron microscopy evidencing the epitaxial growth of ZnMnO_3_ nanosheets on the lateral surfaces of ZnO nanowires at least at early stages of electrodeposition. This highlights the great potential of electrodeposition in the synthesis of metastable phases.

## Experimental

### Electrodeposition of ZnO Nanowires

ZnO nanowire arrays were electrodeposited onto the conducting substrate following the synthesis approach developed by Tena‐Zaera et al.[^35^, ^36^] (for details on the deposition of AZO (aluminum‐doped zinc oxide, ZnO:Al) onto FTO (fluorine‐doped tin oxide, SnO_2_:F)‐coated glass substrates see ESI). For this purpose, a three‐electrode configuration consisting of the AZO/FTO‐coated glass substrate as working electrode, a flat Pt spiral as counter electrode and an Ag/AgCl (1 M KCl) reference electrode (PalmSens) was used. The counter electrode was placed 2.0 cm from the working electrode. The AZO/FTO‐substrate was covered with Teflon tape to define the area (2.25 cm^2^) exposed to the electrolyte. ZnO nanowire deposition was performed at 80 °C in an oxygen (O_2_ 5.0)‐purged 1 M KCl (Sigma Aldrich, purity ≥99.0 %) and 0.5 mM ZnCl_2_ (Sigma Aldrich, anhydrous, purity ≥98 %) aqueous solution (*V*=135 mL). An electrodeposition potential *E*=−1.026 V vs. Ag/AgCl (1 M KCl) reference electrode was applied until a total charge of 14.0 C cm^−2^ had passed. The resulting ZnO nanowire array was thoroughly rinsed with ultrapure water and dried at room temperature in air.

### Electrodeposition of ZnMnO_3_

The electrodeposition of ZnMnO_3_ was performed in a three‐electrode configuration, where a ZnO nanowire or nanoparticle electrode was used as the working electrode, a flat Pt spiral aligned parallel to the working electrode acted as the counter electrode and an Ag/AgCl (1 M KCl) electrode (PalmSens) was used as the reference electrode. A nitrogen (N_2_ 5.0)‐purged 0.175 mM KMnO_4_ (Sigma Aldrich, purity ≥99 %) aqueous solution (*V*=80 mL) was used as the precursor for ZnMnO_3_ deposition and as the electrolyte.

### Electrochemical Characterization

Electrochemical characterization of ZnO and ZnMnO_3_/ZnO electrodes was carried out with a computer‐controlled Autolab PGSTAT302 N potentiostat (Metrohm). Measurements were performed in a three‐electrode cell using a platinum wire as the counter electrode and an Ag/AgCl (3 M KCl) reference electrode (BasInc). N_2_‐purged 1.0 M Na_2_SO_4_ aqueous solution was used as the electrolyte.

Cyclic voltammograms were recorded at a scan rate *v*=0.020 Vs^−1^ in a potential window −0.1 V≤*E*≤1.0 V. For the sake of comparability, potentials applied upon electrodeposition of ZnO and ZnMnO_3_ as well as upon electrochemical characterization are referred to an Ag/AgCl (3 M KCl) RE throughout the paper and will be indicated as *E*_Ag/AgCl_.

The specific capacitance *C* was extracted from cyclic voltammograms as recorded with a sweep rate *v* in the potential range *E_0_* ≤*E*≤*E_1_* (and *E_1_*–*E_0_* ≡Δ*E*) by using Equation 1^[37]^

(1) $$C=\frac{1}{2\cdot v\cdot \Delta E\cdot m}\int_{E_{0}}^{E_{1}}\left(i_{a}+\left|i_{c}\right|\right)dE$$

where *i_a_* and *i_c_* is the current of the anodic and cathodic branch of the cyclic voltammogram, respectively, and *m* is the electrode mass. Unless otherwise stated, the electrode mass is calculated as the sum of the masses of the electroactive phase (i. e. ZnMnO_3_) and of the porous substrate (i. e. the ZnO nanowire array or random nanoparticle network), respectively.

### Spectroscopic and Microscopic Sample Characterization

Scanning electron micrographs were recorded on a Zeiss Gemini Ultra 55 scanning electron microscope (SEM), which is equipped with a field emission gun. The acceleration voltage was set to 3 kV for all samples to avoid charging. The working distance was set between 2.8 and 5.0 mm. All scanning electron micrographs were recorded with an in‐lens secondary electron detector.

A JEOL F200 (scanning) transmission electron microscope (STEM/TEM) equipped with a cold field emission gun and operated at 200 kV was employed to record (high resolution) transmission electron micrographs on a TVIPS F216 CMOS camera (2k×2k).

The natural lattice misfit between epitaxial overlayers and the substrate was determined according to

(2) $$f=\frac{a_{s}-a_{l}}{a_{l}}$$

where $a_{s}$ is the relaxed lattice parameter of the substrate and $a_{l}$ is the relaxed lattice parameter of the overlayer, respectively.^[38]^ The coincidence lattice misfit was calculated based on

(3) $$F=\frac{{na}_{s}-{ma}_{l}}{{ma}_{l}}$$

where m and n are positive integers.^[39]^

A windowless JEOL Centurio energy‐dispersive X‐ray detector (100 mm^2^, 0.97 srad, energy resolution <133 eV) contained within the transmission electron microscope was used for energy‐dispersive X‐ray (EDX) spectroscopic analysis. Measurements were performed with a beam current of 0.1 nA and a beam diameter of 0.16 nm. Elemental maps consist of 256×256 pixels and the integration time of each pixel was 10 μs. At least 700 recorded frames were overlaid for every elemental map resulting in a total acquisition time ≥7 min.

UV/Vis spectra of the immobilized films were recorded with a PerkinElmer LAMBDA 1050 UV/Vis/NIR spectrophotometer equipped with a 150 mm integrating sphere.

## Results and Discussion

### Reactivity of Native Defects in Electrodeposited ZnO Nanowires towards MnO_4_^−^

**Figure 1:**
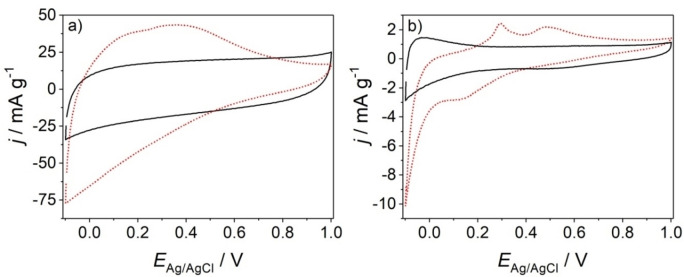
Black, solid lines: Cyclic voltammograms of a pristine ZnO nanowire electrode (a) and of a ZnO nanowire electrode after thermal annealing at 600 °C for 1 h (b). Red, dotted lines: Cyclic voltammograms of the same electrodes after their storage (for 2 h) in a N_2_‐purged 0.175 mM KMnO_4_ aqueous solution at open circuit conditions in the dark. Electrolyte: 0.1 M Na_2_SO_4_ aqueous solution purged with N_2_; *v*=0.020 V s^−1^.

The cyclic voltammogram of a pristine ZnO nanowire electrode in 0.1 M Na_2_SO_4_ features in the potential range −0.1 V≤*E*_Ag/AgCl_≤1.0 V a distorted rectangular shape (Figure 1a). The potential‐independent and reversible capacitive currents are characteristic of double‐layer charging. The electrodeposition approach used in this study[^35^, ^36^] yields ZnO nanowire arrays with a high donor density (*N*_D_ ∼10^19^ to 10^20^ cm^−3^) as evidenced previously by impedance spectroscopy.[^40^, ^41^] The donor density in electrodeposited nanowires is significantly higher than the one determined for ZnO nanowires grown by vapor phase techniques (10^17^–10^18^ cm^−3^).[^42^, ^43^] This high donor density, which was attributed to oxygen vacancies, Zn interstitials or hydrogen,[^40^, ^41^, ^44^, ^45^, ^46^] gives rise to pronounced band bending and thus to a high space charge layer capacitance. The chemical reactivity of charge carriers associated with the n‐type doping of the semiconductor is unexplored so far. Here we raise the question whether these charge carriers can be exploited in interfacial charge transfer reactions thus yielding new synthesis routes for semiconductor nanocomposite materials.

The electrical response of a pristine ZnO nanowire electrode changes significantly after storage for 2 h in a 0.175 mM KMnO_4_ aqueous solution (purged with N_2_) at open circuit conditions in the dark (Figure 1a) and subsequent washing with ultrapure water. A broad current peak with a shoulder at *E*_Ag/AgCl_=0.2 V and a maximum at *E*_Ag/AgCl_=0.4 V is observed in the positive going scan of the cyclic voltammogram (Figure 1a). After reversal of the potential, negative currents are detected. The nearly symmetrical shape of the cyclic voltammogram points to the capacitive origin of the currents. Indeed, an increase of the accumulated charge *q* from 0.45 C ⋅ g^−1^ (for the pristine electrode) to 1.57 C ⋅ g^−1^ (for the electrode treated in KMnO_4_ solution) is determined by integration of the anodic branch of the corresponding voltammogram and division by the scan rate *v*.^[47]^ Obviously, soaking in KMnO_4_ solution and subsequent washing with ultrapure water leads to an increased capacitance of pristine ZnO nanowire electrodes. At the same time, capacitive currents at *E*_Ag/AgCl_≥0.8 V are lower for KMnO_4_‐treated electrodes than for pristine electrodes. This points to a decrease of the donor density in the semiconductor upon KMnO_4_‐treatment and thus to an interfacial electron transfer from the n‐type doped ZnO to MnO_4_^−^. Obviously, one part of the electrons associated with donor states such as oxygen vacancies, Zn interstitials or hydrogen in ZnO nanowires are reactive towards the strong oxidative agent MnO_4_^−^. More importantly, this electron transfer and the reduction of MnO_4_^−^ are associated with the formation of surface deposits imparting to the electrode a pseudocapacitve behavior.

It was shown previously that a thermal annealing of electrodeposited ZnO nanowire arrays at *T*=450 °C in air decreases the donor density by two orders of magnitude.^[41]^ A decrease of the donor density gives rise to a reduction of the space charge layer capacitance.^[41]^ Consequently, two different contributions to the total capacitance of thermally annealed ZnO nanowire electrodes were identified. If the electrode was biased into strong depletion (i. e. at highly positive potentials), the total capacitance resulted primarily from the capacitance of the (uncovered) ZnO seed layer. Only under weak depletion the space charge layer capacitance of ZnO nanowires contributes significantly to the total capacitance.^[41]^ The cyclic voltammogram of ZnO nanowire arrays annealed at 600 °C (Figure 1b) is perfectly in line with these previous findings. The low constant current density observed at 0.2 V≤*E*_Ag/AgCl_≤1.0 V originates from the double layer capacitance of the dense AZO layer. At potentials (*E*_Ag/AgCl_<0.2 V) an increase in current density indicates the contribution of the space charge capacitance from ZnO nanowires. However, the capacitive currents (as well as the capacitance) are much lower after thermal annealing (Figure 1b) as compared to a pristine electrode (Figure 1a). Specifically, a decrease of the accumulated charge *q* from 0.45 C ⋅ g^−1^ (for the pristine electrode) to 0.05 C ⋅ g^−1^ (for the thermally annealed electrode) is observed. Also after a KMnO_4_‐treatment, capacitive currents remain very low (Figure 1b). However, capacitive current peaks at *E*_Ag/AgCl_=0.30 V and 0.48 V as well as at *E*_Ag/AgCl_=0.12 V are detected, respectively, in the positive‐ and negative‐going scans of the cyclic voltammogram.

The results evidence that electrons associated with defects formed upon low‐temperature electrodeposition of ZnO nanowires are reactive and undergo interfacial transfer to MnO_4_^−^ under open circuit conditions in the dark. However, the amount of deposit is limited by the number of reactive electrons. We are therefore aiming at increasing the number of reactive electrons and thus the amount of deposit by an electrochemical i. e. bias‐induced electron accumulation in ZnO nanowire arrays.

### Bias‐induced Interfacial Electron Transfer from ZnO Nanowires to MnO_4_^−^

Electron transfer from a semiconductor to an appropriate acceptor in solution does not necessarily require the application of electrode potentials (*E*_bias_) equal to or more negative than the flat band potential (i. e. *E*_bias_≤*E*_fb_).[^48^, ^49^, ^50^, ^51^] It may also take place at potentials more positive than the flat band potential (i. e. *E*_bias_≥*E*_fb_) by electron tunneling^[52]^ or from mid band gap states associated with defects.[^51^, ^53^]

**Figure 2:**
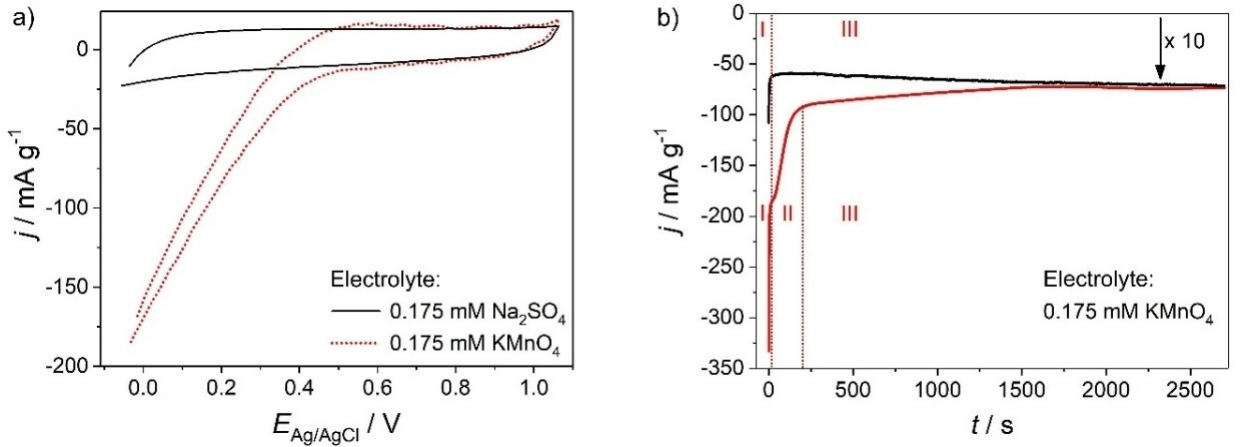
(a) Cyclic voltammograms of a pristine ZnO nanowire electrode. Electrolyte: 0.175 mM Na_2_SO_4_ (black, solid line) and 0.175 mM KMnO_4_ (red, dotted line) aqueous solutions purged with N_2_; *v*=0.020 V s^−1^. The voltammogram recorded in 0.175 mM Na_2_SO_4_ solution was shifted by 45 mV towards more positive potentials to account for the pH‐dependence of the semiconductor band edge potentials.^[54]^ (b) Chronoamperometric profiles measured for pristine ZnO nanowire electrodes upon stepping the electrode potential (from open circuit) to the electrodeposition potential *E*_Ag/AgCl_=0.000 V (red line) or *E*_Ag/AgCl_=0.376 V (black line), respectively. Electrolyte: 0.175 mM KMnO_4_ aqueous solution purged with N_2_. Region I: current spike assigned to the capacitive charging of the semiconductor (reduction of the depletion layer width); Region III: steady state faradaic current. For electrodeposition at *E*_Ag/AgCl_=0.000 V an additional regime of decaying faradaic current is observed (region II) and associated with the depletion of MnO_4_^−^ in the interface‐near region due to the high electron transfer rate (diffusion‐limitation). The current density upon electrodeposition at 0.376 V is multiplied by a factor of 10 for better conspicuity.

To identify an appropriate potential for the electrodeposition of a MnO_4_^−^‐derived phase at the surface of ZnO nanowires, we recorded cyclic voltammograms of a pristine electrode first in 0.175 mM Na_2_SO_4_ aqueous solution (pH 6.0±0.2) and then in 0.175 mM KMnO_4_ aqueous solution (pH 5.3±0.2) (Figure 2a).

The cyclic voltammogram of a pristine ZnO nanowire electrode in 0.175 mM Na_2_SO_4_ features in the entire potential range (‐0.1 V≤*E*_Ag/AgCl_≤1.0 V) a distorted rectangular shape characteristic of space charge layer charging (Figure 2a). The electrical response in 0.175 mM KMnO_4_ resembles the one in 0.175 mM Na_2_SO_4_ at potentials *E*_Ag/AgCl_≥0.50 V, but deviates at *E*_Ag/AgCl_≤0.50 V due to the appearance of a faradaic current. This current results from the interfacial electron transfer from ZnO to MnO_4_^−^. Scanning the electrode potential towards more negative values reduces the space charge layer width and increases the electron concentration in the depletion layer thus increasing the probability for electron tunneling.^[52]^

Electrodeposition was performed (in 0.175 mM KMnO_4_ aqueous solution purged with N_2_) at two different potentials namely at *E*_Ag/AgCl_=0.376 V (i. e. very close to the onset potential of faradaic currents at *E*_Ag/AgCl_=0.500 V) and at *E*_Ag/AgCl_=0.000 V for different electrodeposition times. Chronoamperometric profiles recorded at these two potentials (Figure 2b) contain 2 (electrodeposition at 0.376 V) or 3 (electrodeposition at 0.000 V) distiguishable regions. Immediately after stepping the electrode potential (from open circuit) to *E*_Ag/AgCl_=0.000 V a high current spike is observed (region I), which can be attributed to the capacitive charging of the semiconductor (reduction of the depletion layer width). The initial spike is followed by a region of almost constant current density (region III) pointing to a stabilized faradaic reaction i. e. the interfacial electron transfer to the acceptor species in solution (MnO_4_^−^). For electrodeposition at *E*_Ag/AgCl_=0.000 V an additional regime of decaying faradaic current is observed (region II) and associated with the depletion of MnO_4_^−^ in the interface‐near region due to the high electron transfer rate (diffusion‐limitation). Again, in region III a steady state faradaic current is observed, which is by a factor of ∼10 higher than the one measured at *E*_Ag/AgCl_=0.376 V. Minor variations of the stabilized current density in region III are possibly associated with side reactions (e. g. electron transfer to residual dissolved O_2_) and/or to a slight modification of the electron transfer rate upon the progressive coverage of the ZnO surface by the newly formed deposit.

The total charge, which passed during 120 min accounts for 28±4 C g^−1^ and 600±120 C g^−1^ if electrodeposition was performed at *E*_Ag/AgCl_=0.376 V or 0.000 V, respectively. At the latter potential, the charge increases approximately linearly with deposition time for *t*≤45 min and deviates from linear behavior thereafter (Table 1).

**Table 1:** Total charge, which passed during electrodeposition at *E*_Ag/AgCl_=0.000 V for various deposition times.

time/[min]	total charge passed/[C g^−1^]
10	70±20
45	260±60
120	600±120

### Morphology, Composition and Structure of Electrodeposits

The growth mechanism of ZnO nanowires upon electrodeposition in oxygen‐saturated KCl‐ and ZnCl_2_‐containing aqueous solution involves the reduction of dissolved oxygen at the electrode surface. The associated generation of hydroxide ions induces a local increase of the pH leading to the precipitation of ZnO at the interface.[^36^, ^55^] The grains of the ZnO seed layer act as nucleation sites for nanowire growth.^[35]^ The strongly anisotropic growth of nanowire single‐crystals along the [0001] direction is favored by the internal structure of ZnO^[56]^ and depends furthermore on the local generation rate of hydroxide ions,[^36^, ^40^] which is influenced by the adsorption of chloride ions at the ZnO surface.

**Figure 3:**
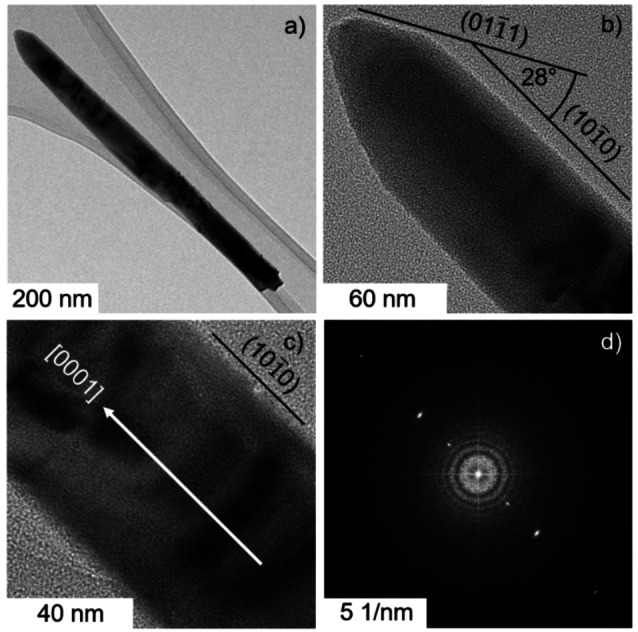
(a) Transmission electron micrograph and (b,c) high resolution TEM images of a ZnO nanowire. The assignment of the^[1]^ direction in wurtzite ZnO is based on the analysis of the FFT pattern (d), which was generated from the micrograph in (c). Indexing of crystallographic planes is based on the analysis of observed angles.

X‐ray diffraction patterns of AZO/FTO‐substrates before and after electrodeposition (Figure S1) indicate that both the sputtered AZO seed layer as well as nanowire arrays feature the hexagonal wurtzite structure and are strongly textured. Specifically, the high intensity of the (0002) diffraction peak at 2θ=34.5° reveals that both the grains of the dense seed layer as well as the nanowires are preferentially orientated with their c‐axis normal to the substrate. This is confirmed by scanning electron microscope (SEM) analysis (Figure S2a), which yields furthermore an average length and diameter of the nanowires of 700 nm and 40 nm, respectively (Figure S3). Nanowires feature smooth lateral facets and hexagonal shape as discernible from the SEM images of the cross section (Figure S2a) and of the top view (Figures S2c) of the electrodeposited films, respectively. The nanowire top region frequently features pyramidal ends (Figures S2a, 3a,b and S4a). It is observed by transmission electron microscopy (TEM) that the crystal planes building up these sharp tips are often tilted by 28° from the [0001] growth direction (Figure 3a,b), which is consistent with ZnO {10$\bar{1}$ 1} planes.[^57^, ^58^] High‐resolution TEM images reveal that nanowires are single crystals as lattice fringes corresponding to the (0001) lattice plane are discernible over the whole nanowire length (Figure 3b,c) and clearly visible in the FFT pattern (Figure 3d). A corresponding lattice spacing of ∼0.528 nm is in good agreement with the c parameter of the ZnO wurtzite phase and allows for an indexing of the lateral facets as {10$\bar{1}$ 0} planes in line with previous reports.[^58^, ^59^]

Elemental maps reveal the presence of chloride ions at the surface of as‐grown ZnO nanowires (Figure S4b–d).

No significant changes are visible in the SEM images of nanowire films after electrodeposition for 120 min at *E*_Ag/AgCl_=0.376 V (Figures S2b,d). However, small deposits at the ZnO surface give rise to a roughening of the nanowires’ lateral faces as well as of the top regions as discernible in TEM images (Figure S5). Elemental maps evidence that the observed roughening results indeed from the deposition of a Mn‐containing phase and is not due to any significant dissolution of ZnO nanowires (Figure S6).

**Figure 4:**
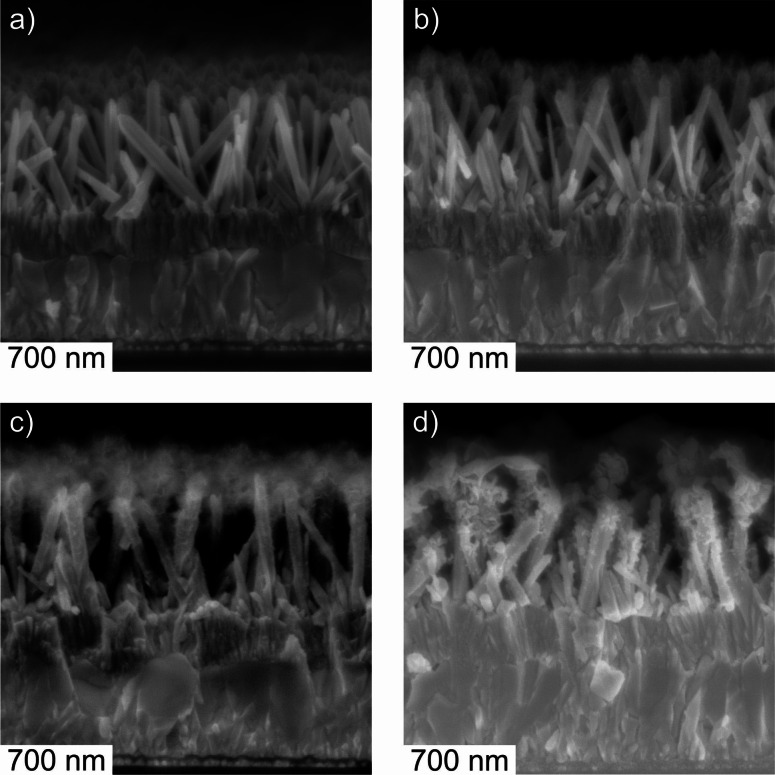
Scanning electron micrographs of the cross section of ZnO nanowire electrodes (a) before and (b–d) after electrodeposition at *E*_Ag/AgCl_=0.000 V in a N_2_‐purged 0.175 mM KMnO_4_ aqueous solution. Deposition time: (b) 10 min, (c) 45 min and (d) 120 min.

**Figure 5:**
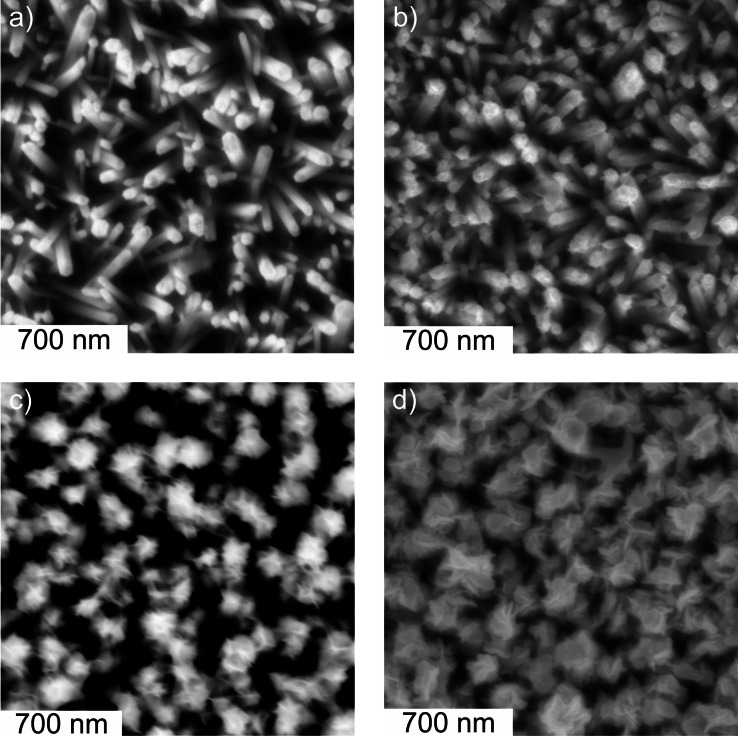
Scanning electron micrographs of the top view of ZnO nanowire electrodes (a) before and (b–d) after electrodeposition at *E*_Ag/AgCl_=0.000 V in a N_2_‐purged 0.175 mM KMnO_4_ aqueous solution. Deposition time: (b) 10 min, (c) 45 min and (d) 120 min.

Significant changes are observed in the SEM images following electrodeposition at *E*_Ag/AgCl_=0.000 V (Figures 4 and 5). For a deposition time of 10 min, micrographs evidence the presence of deposits mainly in nanowire top regions (Figures 4b and 5b). These deposits grow further upon increasing the deposition time to 45 min (Figures 4c and 5c) and 120 min (Figures 4d and 5d), respectively. At least for deposition times ≤45 min, the original morphology of the nanowires seems to be preserved (Figure 4a–c). After 120 min of electrodeposition a partial disintegration of the nanowires can not fully be excluded at least in the nanowire top regions, which are fully covered by the newly formed phase (Figure 4d).

A sheet‐like morphology of the electrodeposits can be anticipated from SEM images. These structures cover mainly the top regions of the original nanowires. The deposit phase features – in addition to the sheet‐like morphology – areas of increased contrast, which possibly result from a twisting of single sheets or from the overlapping of neighboring sheets (Figure 5c,d). Especially for prolonged electrodeposition (deposition time: 120 min), sheet‐like deposits of neighboring nanowires clearly overlap leading to a loss of porosity (Figure 5d). Such an architecture will disconnect the porous structure of the nanowire film from the electrolyte bulk, when the film is operated in an electrochemical cell, which holds true both for the process of electrodeposition itself and for charge accumulation experiments (see below). Indeed, the growth of a deposit layer featuring low porosity gives a rationale for the observed deviation from the initially linear increase of the total passed charge at electrodeposition times >45 min (Table 1). To avoid significant diffusion‐limitation in the charge accumulation/extraction process due to a loss of (inter‐nanowire) porosity we will limit (unless otherwise stated) further characterization to films resulting from electrodeposition (in KMnO_4_ solution) for 45 min.

**Figure 6:**
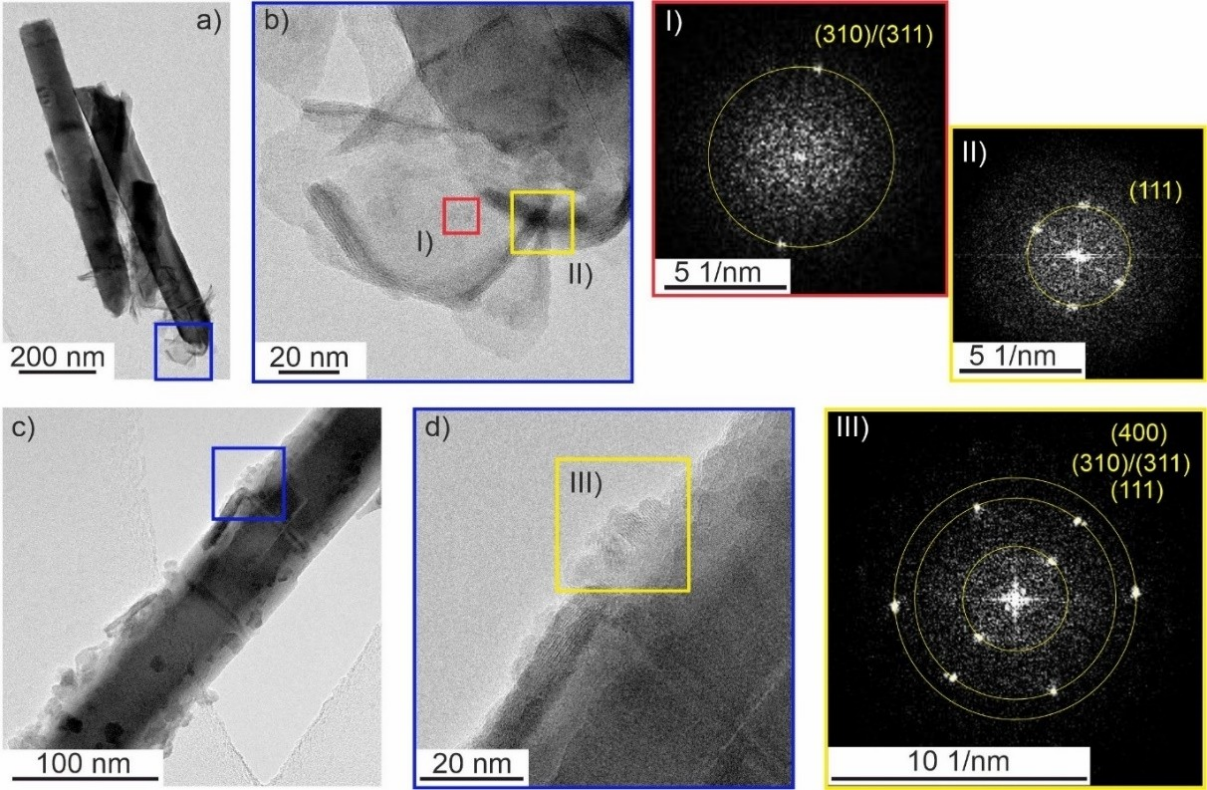
(a,c) Transmission electron micrographs and (b,d) high resolution TEM images (corresponding to sample regions indicated by blue squares in (a) and (c), respectively) of ZnO nanowires following electrodeposition at *E*_Ag/AgCl_=0.000 V in aqueous KMnO_4_ solution (deposition time: 45 min). (I–III) FFT patterns of sample spots indicated by yellow and red squares in (b) and (d). FFT spots are assigned to lattice planes in defective cubic spinel ZnMnO_3_ and organized from low to high real space lattice spacings (see also Table S1).

To better characterize the morphology, crystal structure and composition of the electrodeposits, transmission electron micrographs were recorded after scratching a part of the nanowire‐based film off the substrate. TEM images further corroborate the presence of sheet‐like structures at the surface of ZnO nanowires following electrodeposition (Figures 6, S7 and S8). The newly formed deposit is mainly located in the nanowire top region. The sheets feature lateral dimensions of up to 100 nm (Figure 6b) and a thickness of <10 nm (Figure 6b,c,d). As clearly, discernible from high resolution TEM images single nanosheets are twisted giving thus rise to regions of increased contrast (Figure 6b) in line with SEM observations. In addition to the very prominent sheet‐like structures, TEM images also reveal the presence of particle‐type deposits featuring particle sizes ≤10 nm (Figure 6c,d). High‐resolution TEM images evidence that the newly formed phase is crystalline as lattice fringes are discernible for all (sheet‐like and particle‐type) deposits (Figure 6b,d) and clearly visible in the FFT patterns (Figure 6 I–III). The corresponding lattice spacings (Table S1) are in good agreement with the defective cubic spinel structure of ZnMnO_3_.^[14]^ The small extension of the crystallites (at least in one dimension) together with the low amount of the newly formed phase may explain, why the electrodeposits are XRD‐silent (not shown).

**Figure 7:**
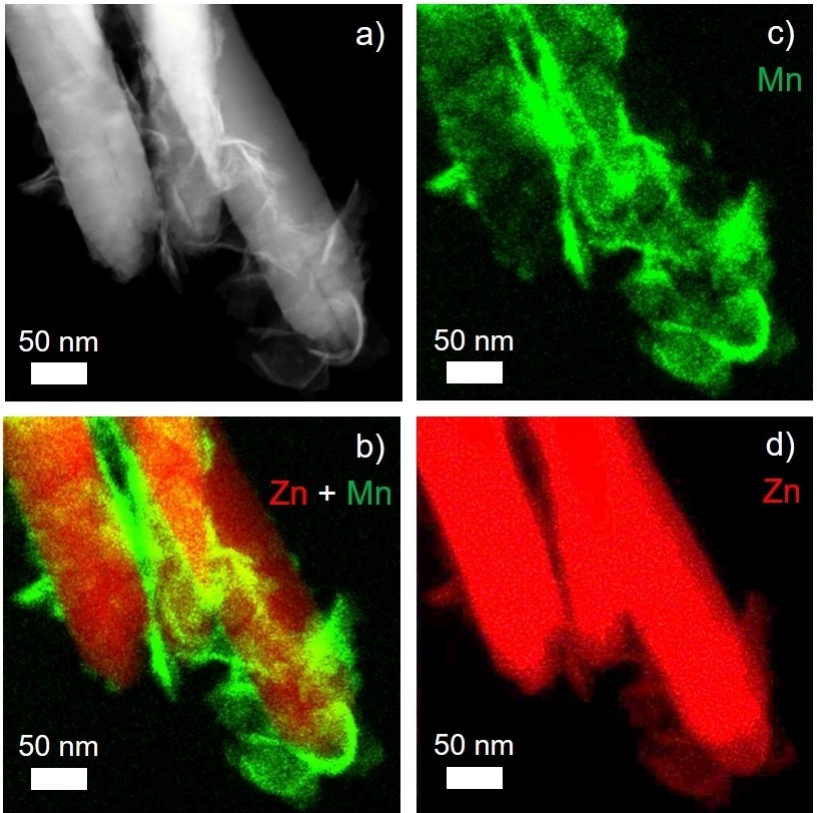
STEM‐HAADF image (a) and elemental intensity maps (b–d) as obtained by EDX analysis of composite nanostructures resulting from electrodeposition at *E*_Ag/AgCl_=0.000 V in KMnO_4_ aqueous solution (deposition time: 45 min). Single elemental maps of Mn (c) and Zn (d) are combined to a mixed elemental map (b).

The elemental composition of the electrodeposits was investigated by EDX analysis. A representative EDX spectrum of the deposit phase contains – in addition to carbon, gold and silicon signals, which originate from the TEM grid (Au‐supported lacey carbon) and the silicon drift EDX detector, respectively – the contributions from three main elements i. e. zinc, oxygen and manganese (Figure S9). The decoration of the top regions of ZnO nanowires is clearly visible in STEM high angle annular dark field (HAADF) images (Figures 7a and S8). Both zinc and manganese are contained in the newly formed phase as visible both from the elemental map of the composite (Figures 7c,d) and from the EDX spectrum of the electrodeposit (Figure S9). The quantification of Mn and Zn present in the electrodeposited phase yields an approximate Mn to Zn atomic ratio of 0.8 : 1.0 (Figure S10). The elemental ratio of Mn : Zn of roughly 1 : 1 and the lattice spacings deduced from FFT patterns (Table S1) evidence the synthesis of a composite structure by electrodeposition of the defective cubic ZnMnO_3_ phase onto the surface of ZnO nanowires at room temperature.

### Epitaxial Growth of ZnMnO_3_ on the Lateral Facets of ZnO Nanowires

**Figure 8:**
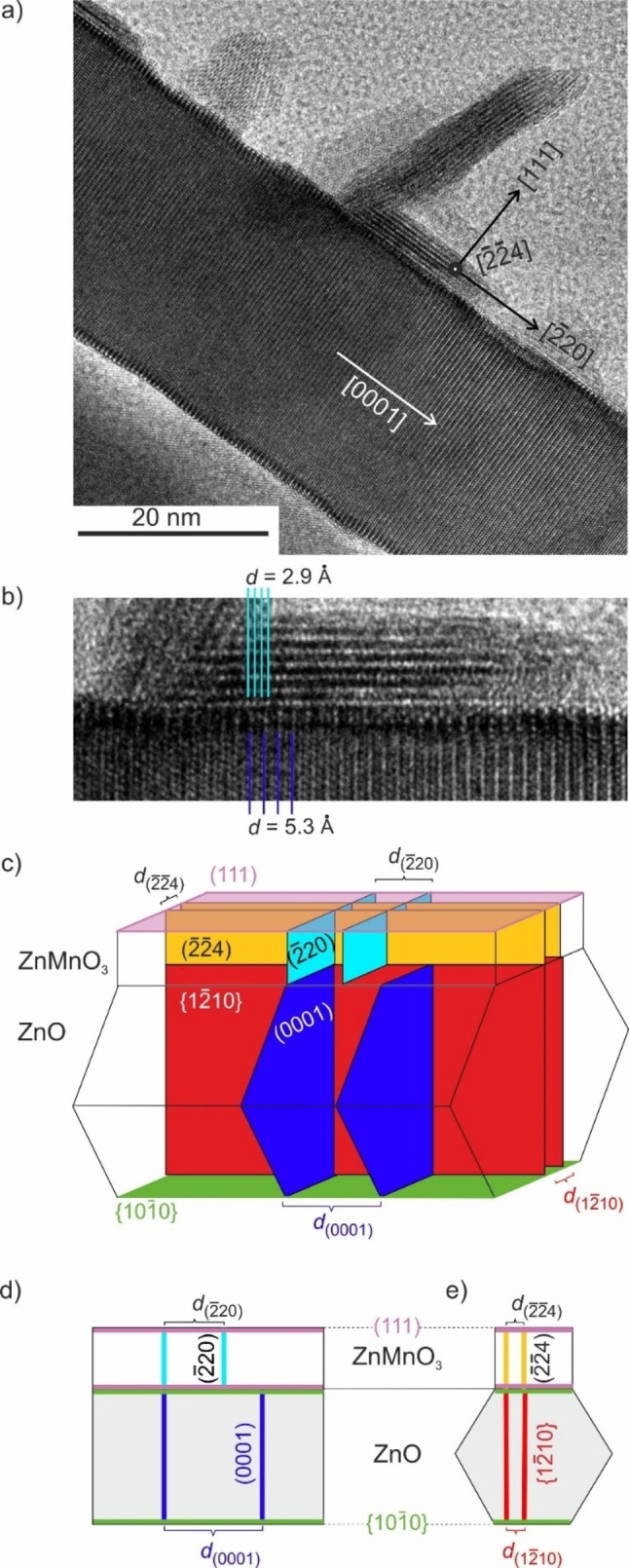
(a) High resolution TEM image of a ZnMnO_3_/ZnO composite and magnification of the sample region featuring the solid/solid interface (b). Assignment of crystallographic directions is based on an in depth analysis of high resolution TEM images and FFT patterns obtained thereof (see Figure S11). (c–e) Schematic representations of a cubic ZnMnO_3_ nanosheet grown on a hexagonal ZnO nanowire. Crystallographic planes relevant for epitaxial growth are indicated together with the respective interplanar spacings.

Proper alignment of composite structures in the electron beam of the transmission electron microscope yields TEM images, which feature under appropriate conditions lattice fringes of both phases i. e. hexagonal wurtzite ZnO and defective cubic spinel ZnMnO_3_. Selected TEM images have been analyzed in detail to gain a deeper insight into ZnMnO_3_ growth on ZnO nanowires. For this purpose, lattice planes were indexed by evaluating high resolution TEM images. Micrographs and corresponding FFT patterns (Figure S11) reveal that the [111] direction in ZnMnO_3_ is perpendicular to the extended surfaces of the quasi‐2‐dimensional crystals (Figure 8a). Consequently, nanosheets are expected to expose mainly (111) planes at the surface. The separation of lattice fringes perpendicular to the [111] direction (*d*=2.9 Å ) is consistent with the lattice spacing of ($\bar{2}$ 20) planes (Figures 8b and S11, Table S2).^[14]^ By calculating the cross product, we identify the [$\bar{2}\bar{2}$ 4] direction as the crystallographic direction, which is perpendicular to both [111] and [$\bar{2}$ 20] directions, respectively (Figure 8a). From the high resolution TEM images (Figures 8a,b and S11a,b) it can furthermore be deduced that the ZnMnO_3_ (111) plane is parallel to the ZnO (10$\bar{1}$ 0) plane and thus to the [0001] direction. In the hexagonal wurtzite structure the (1$\bar{2}$ 10) plane is perpendicular to both the (0001) plane and to the (10$\bar{1}$ 0) plane.^[60]^ This gives rise to an arrangement of lattice planes as highlighted by the schemes in Figures 8c–e and S12. The corresponding interplanar spacings are listed in Table 2.

**Table 2:** Lattice planes, interplanar spacings and lattice mismatch for epitaxially grown ZnMnO_3_ sheets on ZnO nanowires.

phase	lattice plane	*d*/[Å]	
ZnO	(1$\bar{2}$ 10)	1.6480^[a]^	**lattice misfit/[%]**:
ZnMnO_3_	($\bar{2}\bar{2}$ 4)	1.7034 (Ref. [14])	−3.3
ZnO	(0001)	5.2065 (Ref. [61])	**coincidence lattice misfit/[%]**:
ZnMnO_3_	($\bar{2}$ 20)	2.9503 (Ref. [14])	−11.8

^[a]^ Calculated value^[62]^ using the lattice parameters of hexagonal ZnO: a=3.296 Å and c=5.2065 Å .^[61]^

Based on the assignment of lattice planes, epitaxial growth of ZnMnO_3_ nanosheets on {10$\bar{1}$ 0} planes of wurtzite ZnO is anticipated. However, for epitaxial growth to occur, there must be a good fit of lattice spacings and orientation between the deposit and the substrate. Indeed, we observe a very small misfit of −3.3 % between the lattice spacings of ZnMnO_3_ ($\bar{2}\bar{2}$ 4) and ZnO (1$\bar{2}$ 10). However, the misfit between the lattice spacings of ZnMnO_3_ ($\bar{2}$ 20) and ZnO (0001) is very large (76 %). Epitaxial growth in large‐misfit systems must be connected with an interface configuration exhibiting a low total energy.^[63]^ In such a case, an epitaxial interface described by a so‐called coincidence lattice may form, if film and substrate feature lattice spacings close to an integer ratio m/n.^[63]^ Indeed, for m/n=2 a moderate coincidence lattice misfit of −11.8 % can be determined between the lattice spacings of ZnMnO_3_ ($\bar{2}$ 20) and ZnO (0001), which may explain the observed epitaxial growth at least for thin deposit structures (Figures 8a,b and S11a,b). Epitaxial growth will continue until the strain, which originates from the lattice mismatch cannot be sustained any longer. Accordingly, delamination of epitaxially grown ZnMnO_3_ should occur along the direction, which is associated with the larger lattice mismatch, i. e. along the c‐axis of the ZnO nanowires. Indeed, such a behavior is observed in many different sample regions (Figures 8a, S11a and S13). The partial detachment may be followed by a significant twisting of the nanosheets as a consequence of their two‐dimensional morphology. Epitaxial growth of two phases with a significant lattice mismatch at least in one crystallographic direction was observed before, for instance, for δ‐Bi_2_O_3_ on ZnO nanowires^[60]^ and for WS_2_ on ZnO.^[64]^

The two‐dimensional morphology of electrodeposited ZnMnO_3_ resembles the morphology of some electrodeposited MnO_2_ nanostructures.[^65^, ^66^] For instance, layered manganese oxides (δ‐MnO_2_) have been synthesized from aqueous KMnO_4_ solutions using cathodic reduction.[67–70] In δ‐MnO_2_, two‐dimensional sheets consisting of edge‐sharing [MnO_6_] octahedra are stacked to form a layered material. These sheets are stabilized by cations and water located in between the layers thus forming two‐dimensional crystalline structures. In previous studies, an inert substrate (Au, Ni) was used for electrodeposition. In the present study, in contrast, the substrate for electrodeposition (ZnO) acts at the same time as a precursor supplying Zn^2+^ ions for ZnMnO_3_ formation. According to a previous X‐ray absorption study, manganese ions (Mn^3+^, Mn^4+^) in ZnMnO_3_ present octahedral coordination whereas the Zn^2+^ ions are tetrahedrally coordinated.^[17]^ The ZnMnO_3_ structure (defective cubic spinel, space group Fd$\bar{3}$ m)^[17]^ can therefore be thought of being built up from honeycomb‐like layers of edge sharing octahedral sites extending the (111) plane. These layers are stagged along the [111] direction and interconnected by corner sharing isolated tetrahedral (fitting into the pseudo‐hexagonal interstitials of the (111) octahedral layers) and edge sharing octahedral sites. By this, a 3‐dimensional interconnected structure is obtained, where octahedral [MnO_6_] chains extend in 4 different <1$\bar{1}$ 0> directions, the interstitials are occupied by the isolated tetrahedrally coordinated [ZnO_4_] sites.

Importantly, high resolution transmission electron micrographs (Figure 8) indeed evidence the growth of ZnMnO_3_ nanosheets at ZnO {10$\bar{1}$ 0} facets by a stacking of layers in the [111] direction. Whether the growth of the nanosheets in the [111] direction is associated with a dissolution‐(re)crystallization mechanism or whether it is connected to the diffusion of Zn^2+^ ions from the buried ZnO/ZnMnO_3_ interface along the [111] direction to the reactive ZnMnO_3_/electrolyte interface is unknown so far. However, Zn^2+^ diffusion in the emerging structure may be facilitated by the fact that in the spinel structure [ZnO_4_] tetrahedra are connected by empty octahedra forming a percolating network in three dimensions and leading to a unique diffusion topology in the spinel framework.^[71]^ Importantly, reversible Zn^2+^ intercalation has been observed in cation‐defective ZnMn_2_O_4_ in aqueous media.^[72]^ The unique mechanism of ZnMnO_3_ formation based on the electrochemical reduction of solution species at the surface of a reactive substrate acting as the Zn precursor may allow for tuning the cation non‐stoichiometry in the electrodeposited phase by minute control of the process parameters. This would be of great importance in the search for advanced electrode materials in multivalent ion batteries.[^71^, ^72^]

It is evident from transmission electron micrographs (Figures 6, 8 and S13) that very smooth ZnO {10$\bar{1}$ 0} facets are required for epitaxial nanosheet growth to take place. In contrast, at rough surface regions particle‐like ZnMnO_3_ morphologies are observed (Figures 6c,d and 8a).

### Optical Properties of ZnMnO_3_

**Figure 9:**
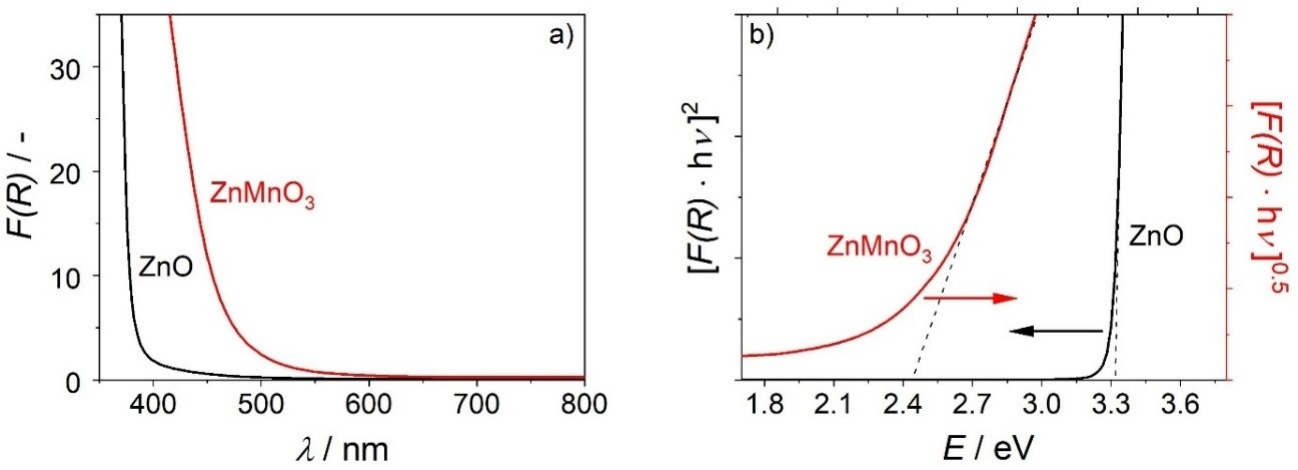
(a) UV/Vis diffuse reflectance spectra and (b) Tauc plots of a ZnO nanowire electrode (black lines) and of a ZnMnO_3_/ZnO nanocomposite film (red lines). Linear regions in the Tauc plots are fitted (dashed lines) and extrapolated to get an estimate of the band gap energy.

Figure 9a shows Kubelka−Munk functions *F(R)* derived from UV/Vis diffuse reflectance spectra of pristine ZnO nanowire arrays and ZnMnO_3_/ZnO nanocomposite films. The data reveal absorption thresholds at *E* ∼3.3 eV (*λ*=376 nm) and 2.4 eV (*λ*=508 nm), respectively, as extracted from the corresponding Tauc plots (Figure 9b). Both ZnO^[61]^ and ZnMnO_3_ ^[73]^ are semiconductors. For Tauc‐plots based on diffuse reflectance data, (*F(R)* ⋅ *hν*)^*n*^ is plotted against the photon energy (*hν*). For direct semiconductors, the exponent *n* takes a value of 2, for indirect semiconductors *n*=0.5. Extrapolation of the linear region in Tauc plots and analysis of the *E*‐axis intercepts gives an estimate of the band gap energy.^[74]^ For ZnO, a well‐known direct semiconductor, *n*=2. The extracted band gap energy of ZnO nanowires (*E_BG_* =3.3±0.1 eV) is in good agreement with literature (*E_BG_* =3.37 eV).^[61]^ Based on the assumption that ZnMnO_3_ is an indirect semiconductor, a band gap energy *E_BG_* =2.4±0.1 eV is extracted from the Tauc plot of the ZnMnO_3_/ZnO nanocomposite film.

### Capacitive Behavior of ZnMnO_3_/ZnO Composite Electrodes

**Figure 10:**
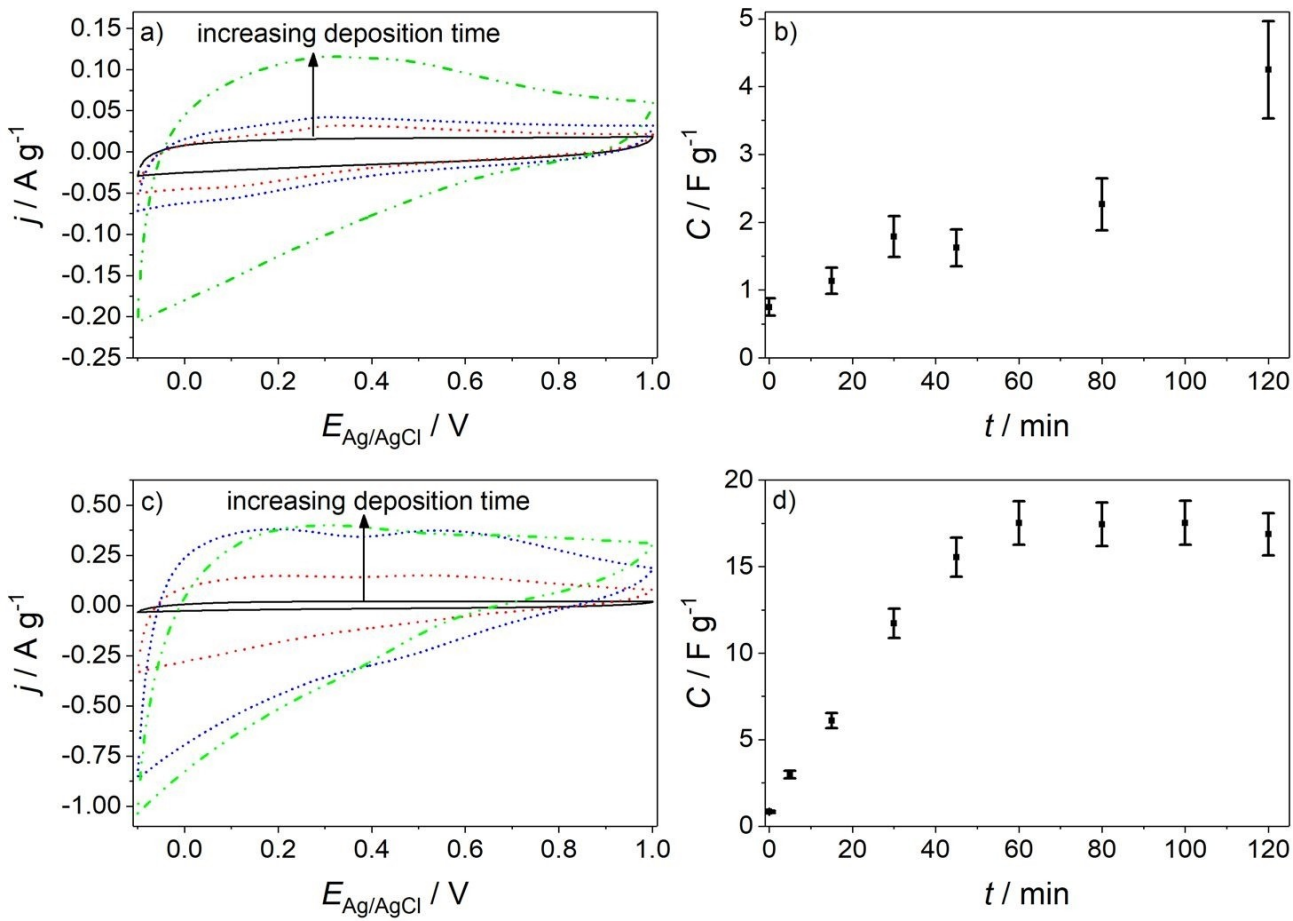
(a,c) Cyclic voltammograms recorded at a scan rate *v*=0.020 V s^−1^ in nitrogen‐purged 1 M Na_2_SO_4_ aqueous solution for ZnO nanowire electrodes before (black, solid lines) and after sequential deposition of ZnMnO_3_ at an electrodeposition potential of (a) *E*_Ag/AgCl_=0.376 V or (c) *E*_Ag/AgCl_=0.000 V, respectively. Electrodeposition time: 15 min (red, dashed lines), 45 min (blue, dotted lines), and 120 min (green, dashed and dotted lines); electrodeposition solution: N_2_‐purged 0.175 mM KMnO_4_ aqueous solution. After each electrodeposition step electrodes were washed with ultrapure water before recording voltammograms in 1 M Na_2_SO_4_ aqueous solution. (b,d) Specific capacitance (as calculated from data in (a) and (c)) as a function of deposition time at an electrodeposition potential of (b) *E*_Ag/AgCl_=0.376 V or (d) *E*_Ag/AgCl_=0.000 V, respectively. The specific capacitance is referenced to the total electrode mass (i. e. the mass of ZnO nanowires and of the ZnMnO_3_ deposit). The specific capacitance of a ZnO nanowire electrode accounts for 0.75±0.20 F g^−1^.

Cyclic voltammograms in nitrogen‐purged 1 M Na_2_SO_4_ aqueous electrolyte (Figure 10a,c) were recorded (in the potential range −0.1 V≤*E*_Ag/AgCl_≤1.0 V) for pristine ZnO nanowire electrodes and after electrodeposition of ZnMnO_3_ (ZnMnO_3_/ZnO composite electrodes) at *E*_Ag/AgCl_=0.376 V (Figure 10a) or at *E*_Ag/AgCl_=0.000 V (Figure 10c), respectively. Deposition of ZnMnO_3_ leads for both electrodeposition potentials to significant changes in the voltammograms. The positive going scans feature a broad contribution of positive current in addition to the capacitive response resulting from ZnO nanowires. Negative currents are detected upon a reversal of the potential. The virtual matching of the charge accumulated in the electrode in the positive going scan and of the charge extracted from the electrode upon potential‐reversal highlights the capacitive origin of the detected currents and evidences the possibility of reversible charge accumulation in ZnMnO_3_. The shape of the cyclic voltammograms deviates from the rectangular shape characteristic of ideal electrochemical capacitors pointing to the presence of pseudocapacitance in ZnMnO_3_.^[75]^ In contrast, nearly rectangular shapes have been observed in aqueous electrolyte for nanostructured birnessite δ‐MnO_2_, which has been attributed to interlayer cation intercalation involving nanoconfined structural water.^[70]^ Clearly, a different capacitive mechanism contributes to charge storage in ZnMnO_3_.

**Figure 11:**
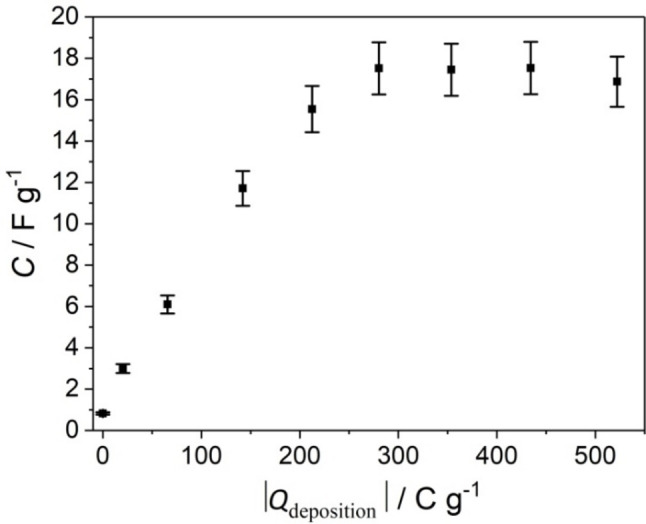
Specific capacitance of ZnO/ZnMnO_3_ composites (as determined by cyclic voltammetry in 1 M Na_2_SO_4_ aqueous solution, compare Figure 10c,d) versus total charge passed during electrodeposition at *E*_Ag/AgCl_=0.000 V in 0.175 mM KMnO_4_ aqueous solution. The specific capacitance of a ZnO nanowire electrode accounts for 0.75±0.20 F g^−1^.

For electrodeposition of ZnMnO_3_ at *E*_Ag/AgCl_=0.376 V an increase of the deposition time in the range 15 min≤*t*≤120 min leads to a continuous increase of the capacitive current density (Figure 10a) and of the specific capacitance *C* (Figure 10b). For electrodeposition at *E*_Ag/AgCl_=0.000 V, on the other hand, capacitive current density (Figure 10c) and specific capacitance (Figure 10d) level off for deposition times *t*≥45 min. Especially for electrodeposition at *E*_Ag/AgCl_=0.000 V, cyclic voltammogramms contain discernible features. Current peaks at *E*_Ag/AgCl_=0.1 V and *E*_Ag/AgCl_=0.5 V are detected for deposition times *t*≤45 min in the positive going scan. The shape of the voltammograms does not change significantly for *t*≤45 min. However, a distorted shape is observed for *t*=120 min together with a positive shift of the current peaks by ∼0.2 V (Figure 10c). Both observations point to the presence of an internal resistance following long deposition times. The origin of this resistance can be rationalized based on SEM data in Figures 4 and 5. Electrodeposition of ZnMnO_3_ at *E*_Ag/AgCl_=0.000 V for *t*=120 min leads to a local loss of (inter‐nanowire) porosity (Figures 4d and 5d). This may lead to a reduced accessibility of the reactive solid/electrolyte interface, where charge compensation is expected to take place. In such a situation, the ion transport from the bulk electrolyte solution to the interface may limit the rate of the overall charge accumulation process. Alternatively, a low electronic conductivity of the electroactive material may limit electron transport and thus the rate of the overall process especially for extended morphologies such as the ZnMnO_3_ nanosheets observed by transmission electron microscopy (Figures 6b, S7 and S8). Both interpretations are in line with the observed dependence of the specific capacitance, *C*, of the composite electrodes on the total charge passed during electrodeposition at *E*_Ag/AgCl_=0.000 V, *Q*_deposition_ (Figure 11). This charge was obtained by integration of the current density recorded during electrodeposition over time. Assuming that the Coulombic efficiency of ZnMnO_3_ formation does not change significantly with deposition time, an increase of the charge passed during electrodeposition indicates an increase of the deposit mass, which according to Faraday's law is proportional to the total charge. However, as can be clearly observed from Figure 11 the specific capacitance levels off at *Q*_deposition_ ∼300 C g^−1^ (corresponding to a deposition time of 60 min), despite of a further continuous increase of *Q*_deposition_ up to a deposition time of 120 min. From this analysis, it can be deduced that for long deposition times (*t*≥60 min at *E*_Ag/AgCl_=0.000 V) not all of the electrodeposited mass contributes equally to charge accumulation i. e. not all the ZnMnO_3_ is electrochemically active (at least under the conditions investigated). A limitation of the electrodeposition time at *E*_Ag/AgCl_=0.000 V to 45 min, however, will avoid significant diffusion limitation, while assuring a high mass of the electroactive material. In this context, it has to be mentioned that the specific capacitance of a composite electrode is ∼4 times higher if electrodeposition was performed at *E*_Ag/AgCl_=0.000 V for *t*=45 min (*C*=16.0±2 F g^−1^, Figure 10 d) as compared to electrodeposition at *E*_Ag/AgCl_=0.376 V for *t*=120 min (*C*=4.5±0.5 F g^−1^, Figure 10 b). This is perfectly in line with the much lower current flow during electrodeposition at the latter potential (Figure 2b).

To get an estimate for the specific capacitance of the electroactive material (i. e. ZnMnO_3_), we quantified the electrodeposited mass by determining the charge passed during electrodeposition and using Faraday's law. ZnMnO_3_ is a cubic spinel with oxygen and cation deficiency and hence the oxidation state of Mn varies between +III and +IV.^[17]^ However, we approximate the formation of ZnMnO_3_ by assuming a three electron transfer per MnO_4_^−^ unit according to

(4) $$\mathrm{Zn}{}^{2+}+2\;H{}^{+}+\mathrm{MnO}{}_{4}{}^{-}+3\;e{}^{-}\;\to \;\mathrm{ZnMnO}{}_{3}+H{}_{2}O$$

as Mn^4+^ is the prevalent oxidation state in ZnMnO_3_.^[17]^ To assure a Coulombic efficiency (of ZnMnO_3_ formation) of approximately 100 %, experimental conditions where set such that electron transfer to acceptors different from the strong oxidizing agent MnO_4_^−^ can be neglected. Electrodeposition was therefore performed very close to the onset potential of faradaic currents (Figure 2a) i. e. at *E*_Ag/AgCl_=0.376 V. Analysis of the charge passing during electrodeposition for 120 min (and assuming a Coulombic efficiency of 100 %) yields for the investigated ZnO nanowire film an absolute mass of 4.8±0.7 μg of ZnMnO_3_. This (together with the capacitance as extracted from the cyclic voltammogram recorded at *v*=0.020 V s^−1^, Figure 10a) allows to estimate the specific capacitance of ZnMnO_3_ as being 220 F g^−1^. Around 0.4 electrons can be stored per manganese ion in the potential window investigated. The specific capacitance value as referred to the mass of ZnMnO_3_ is comparable to some electrode architectures based on MnO_2_. Ning et al. synthesized crystalline birnessite‐type MnO_2_ sheets with a specific capacitance of 151.1 F g^−1^ (as determined at *j*=1.0 A g^−1^).^[76]^ A specific capacitance of 423.5 F g^−1^ (as determined at *j*=0.5 A g^−1^) was reported for the same active material on ZnO pillars.^[77]^ While capacitance values of electrodeposited ZnMnO_3_ nanosheets reported here are mediocre, one may further exploit the large parameter window offered by the presented electrodeposition approach to optimize the electrodes’ functional properties. Related studies are underway in our laboratory and an extensive electrochemical characterization work of ZnMnO_3_/ZnO nanocomposite films deposited under different conditions (e. g. different pH values of the precursor solution, bias‐induced versus photoinduced generation of reactive electrons…) will be reported elsewhere.

### Impact of Substrate Morphology – ZnO Nanowire Arrays versus Random ZnO Nanoparticle Networks

To complement the results of ZnMnO_3_ electrodeposition on ZnO nanowires and investigate the impact of substrate morphology on ZnMnO_3_ growth, we studied electrodes consisting of a random ZnO nanoparticle network. The porous morphology of the films with an average thickness of 7.6±2.0 μm is visible in SEM images of the electrode cross section (Figure S14a). TEM images of ZnO nanoparticle aggregates scratched off the substrate reveal particles featuring an irregular but approximately equidimensional shape (Figure S14b) and a size between 10–40 nm with a median value of 19 nm (Figure S14c).

Cyclic voltammograms of ZnO nanoparticle electrodes feature monotonously increasing capacitive currents at *E*_Ag/AgCl_<‐0.25 V (Figure S15), which have been attributed to shallow donors originating from Zn interstitials or hydrogen in the oxide,^[78]^ or alternatively, electrons trapped at surface states.^[79]^ In addition, a capacitive peak at *E*_Ag/AgCl_=0.01 V points to the presence of deep traps. Applying an external bias of *E*_Ag/AgCl_=0.000 V thus induces a partial filling of these deep trap states and – provided that electron transfer from these traps to acceptors in solution is possible – allows for the electrodeposition of ZnMnO_3_ on ZnO nanoparticle electrodes at the same potential as on ZnO nanowire electrodes. Indeed, constant electrodeposition currents develop also for ZnO nanoparticle electrodes at this potential.

Cyclic voltammograms of a ZnO nanoparticle electrode, which was subjected to consecutive electrodeposition steps at *E*_Ag/AgCl_=0.000 V, are shown in Figure S16. An increase of the deposition time in the range 15 min≤*t*≤120 min leads to a continuous increase of the capacitive current density (Figure S16a) and of the specific capacitance *C* (Figure S16b). However, the slope of the initial fast increase in capacitance clearly flattens at *t*≥30 min (Figure S16b). Furthermore, a strong distortion of voltammograms is observed for composite electrodes even after short electrodeposition times (Figure S16a), which becomes more and more pronounced upon further increasing the deposition time.

The electrochemical data point to a reduced accessibility of the reactive solid/electrolyte interface after prolonged electrodeposition and consequently significant diffusion limitation upon charge accumulation. The origin of diffusion limitation can be rationalized by analysis of high resolution TEM images, which have been recorded after scratching a part of the nanoparticle‐based film off the substrate. Importantly, we observed sample regions lacking virtually any newly formed phase (Figure S17a,b), while the original ZnO nanoparticle properties (Figure S14b) remain unchanged. In addition, some sample spots corroborate the presence of a new phase following electrodeposition (Figure S17c–f). This phase is located in between (virtually unchanged) ZnO nanoparticles thus filling interparticle pores leading to quite dense sample regions. TEM data thus allow to attribute diffusion limitation to a local loss of (inter‐nanoparticle) porosity. Specifically, we postulate the formation of a dense layer at the outer electrode part (i.e the interface between the porous film and the bulk electrolyte) due to the filling of pores with the newly formed electrodeposit.

The new phase features in addition to a particle‐like morphology (particle size ≤5 nm, Figure S17c,d) also some elongated and sheet‐like structures (Figure S17e,f). The latter point to the formation of twisted nanosheets also on ZnO nanoparticles. However, the lateral size of these nanosheets is significantly smaller (≤20 nm) than in the case of ZnO nanowire arrays (≤100 nm, Figures S7 and S8). In line with observations on ZnO nanowire arrays, electrodeposits are XRD‐silent (Figure S18). Elemental analysis evidences that both zinc and manganese are contained in the newly formed phase as visible from the elemental map of the composite (Figure S19). However, due to the small size and high intermixing of ZnO nanoparticles and the manganese‐containing phase, it was not possible to quantify the Mn to Zn atomic ratio in the electrodeposits for these electrodes.

The surface area of ZnO nanoparticle (NP) films is significantly higher than the surface area of nanowire (NW) films. The higher surface area of ZnO nanoparticle films would allow in principle for (i) the electrodeposition of a higher amount of the electroactive phase, (ii) a larger interfacial area between the conductive scaffold and the electroactive material and (iii) a higher electrode/electrolyte interface, all of which would be beneficial for efficient charge storage in the electrode. However, these potential advantages can not be exploited by the presented electrodeposition approach due to the partial inaccessibility of the porous structure. In addition, high‐temperature synthesis and processing of ZnO nanoparticle powders and porous films result in low donor densities of the scaffold material. This together with the high concentration of particle/particle interfaces, which slow down electron transport,[80–83] makes the random nanoparticle network as investigated here a suboptimal scaffold structure for the electrodeposition of the electroactive phase.

ZnO nanowire films, on the other hand, provide oriented arrays of elongated single crystalline units for directional electron transport along the nanowire axsis[^34^, ^77^, ^84^] as well as highly accessible pores for efficient ion diffusion both during electrodeposition and upon charge storage. In the latter case, accessibility of the electroactive phase for charge compensating ions is crucial.[^34^, ^77^, ^84^] In addition, low‐temperature electrodeposition of ZnO nanowires results in high donor densities providing a high conductivity of the scaffold material, which is essential for the fast charging and discharging of the electrode.[^77^, ^85^, ^86^] In this context, room temperature deposition of the electroactive material (ZnMnO_3_) – as realized in the synthesis approach highlighted in this contribution – allows for preserving a high donor density of the ZnO scaffold. Synthesis routes for crystalline ZnMnO_3_ reported so far, in contrast, involve high temperature process steps.[^14^, ^18^, ^19^, ^20^, ^21^, ^22^, ^23^, ^24^, ^25^, ^26^]

The presented synthetic approach, which is exemplified here for ZnMnO_3_/ZnO heterostructures, may possibly constitute a more general strategy for the room‐temperature deposition of different ternary oxide nanostructures. While this remains to be proven, more mechanistic insights into the electrodeposition process will allow to elucidate process – as well as material‐related prerequisites (e. g. structural and compositional properties of the involved scaffold and deposit materials) for the successful room‐temperature electrodeposition of crystalline ternary oxides. Corresponding studies are currently underway in our laboratory.

## Conclusions

ZnO nanowire arrays constitute a suitable platform for the electrodeposition of the defective cubic spinel ZnMnO_3_ upon interfacial reduction of MnO_4_^−^ in aqueous solution at room‐temperature. Importantly, ZnO surfaces act as the Zn precursor in the formation of ZnMnO_3_. The pronounced 2‐dimensional morphology of the electrodeposit results from the epitaxial growth of ZnMnO_3_ at the extended lateral facets of ZnO nanowire single crystals. Strain originating from the lattice mismatch between the ZnO substrate and the ZnMnO_3_ deposit leads to a partial delamination of epitaxially grown ZnMnO_3_ nanosheets at a later stage of electrodeposition. This results in a high interfacial area between the electroactive material and the electrolyte. At the same time, the electronic connectivity between the conducting substrate and the deposit is preserved. However, prolonged deposition times lead to a local loss of inter‐nanowire porosity, which is detrimental for the capacitive behavior of the ZnMnO_3_/ZnO nanocomposite film leading to significant diffusion‐limitation in the charge accumulation/extraction process.

Our results evidence that the substrate for epitaxial growth represents at the same time the source of one of the metal cations for the interfacial formation of a mixed transition metal oxide. It remains to be elucidated, whether this mechanistic principle is unique to the investigated material combination or may be extended (under appropriate conditions) to other scaffold/deposit systems thus providing a basis for a more general synthesis approach yielding ternary oxide nanostructures.

## Supporting Information

Further experimental details, X‐ray diffractograms of conducting substrates and ZnO nanoparticle films, additional scanning electron and transmission electron micrographs of ZnO and ZnMnO_3_/ZnO films and associated analyses, elemental maps and representative energy dispersive X‐ray spectra. Cyclic voltammograms of ZnO nanoparticles films before and after electrodeposition of ZnMnO_3_.

## Conflict of interest

There are no conflicts of interest to declare.

1

## Supporting information

As a service to our authors and readers, this journal provides supporting information supplied by the authors. Such materials are peer reviewed and may be re‐organized for online delivery, but are not copy‐edited or typeset. Technical support issues arising from supporting information (other than missing files) should be addressed to the authors.

Supporting Information

## Data Availability

The data that support the findings of this study are available from the corresponding author upon reasonable request.
