# Supplementary material for: Substrate‐Enabled Room‐Temperature Electrochemical Deposition of Crystalline ZnMnO3
Source: Chemphyschem. 2022 Oct 25;24(1):e202200586. doi: 10.1002/cphc.202200586 (PMC10092203; doi:10.1002/cphc.202200586)
Supplement: Supplementary file 1 — Supporting Information [file CPHC-24-0-s001.pdf]

ChemPhysChem

Supporting Information

Substrate-Enabled Room-Temperature Electrochemical Deposition of Crystalline $ZnMnO_3$

Karin Rettenmaier, Gregor A. Zickler, Günther J. Redhammer, and Thomas Berger*

1. Further Experimental Details

1.1. Preparation of the Conducting Substrate

FTO (fluorine-doped tin oxide, SnO_2:F)–coated glass substrates (Pilkington TEC 8, resistance 8 Ω/□) were cleaned at 60 °C upon ultrasonication first in aqueous Hellmanex III solution (Hellma Analytics, 2 v%), than in isopropanol (technical grade, Acros chemicals), and finally in ultrapure water (Merck Millipore, 18 MΩ cm). After drying in an oven at 80 °C the substrates were treated in an oxygen plasma (Emitech, K1050X RF Plasma Cleaner). A dense AZO (aluminum-doped zinc oxide, ZnO:Al)–layer was deposited onto the FTO–substrates to facilitate the adhesion of ZnO nanowires grown by electrochemical deposition and guarantee a homogeneous ZnO nanowire coverage.[1] The AZO–layer was sputtered using a Clustex 100 M sputter coater (Leybold Optics) onto the cleaned FTO–substrates (base pressure before sputtering: $1 \cdot 10^{-6}$ mbar, sputter conditions: Ar–flow (Ar 5.0): 20 sccm, power: 250 W, deposition time: 300 s). After sputter deposition, the substrates were annealed at 80°C for 1 h.

1.2. Preparation of ZnO Nanoparticle Electrodes

ZnO nanoparticle powders were prepared by metal organic chemical vapor synthesis (MOCVS) in a hot wall reactor at 15 ± 2 mbar.[2,3] $Zn(CH_3COO)_2 \cdot 2H_2O$ (Sigma Aldrich, purity 99 %) was evaporated and transported in an O_2 gas flow (O_2 5.0) to the reaction zone (T = 800 °C), where decomposition of the Zn-precursor, formation of ZnO nuclei via homogeneous nucleation and successive crystal growth occur. The nanoparticles were collected down-stream in a stainless steel net. The as-synthesized nanoparticle powder was subjected to a thermal treatment under high vacuum conditions and in defined oxygen atmospheres for further product purification. Additional details are provided in references [2,3].

The ZnO powder was used as precursor for the preparation of ZnO electrodes consisting of a mesoporous and random nanoparticle network. A stabilized slurry was prepared from 0.150 g ZnO nanoparticle powder, 10 µL acetylacetone (Sigma Aldrich, purity ≥ 99%), 10 µL TritonX-100 (Sigma Aldrich, laboratory grade) and 0.6 mL ultrapure water. Electrodes were obtained by spreading the slurry onto FTO substrates by doctor blading. The ZnO nanoparticle films were dried in air at room temperature and subsequently annealed at 450°C for 1 h.

1.3. Sample Preparation for TEM Analysis

Sample preparation for TEM analysis consisted of scratching one part (around one third) of the ZnO nanowire array, the random ZnO nanoparticle network or, alternatively, of the $ZnMnO_3$/ZnO composite film off the substrate with a razor blade. The obtained powder was

dispersed in absolute ethanol (800 µL, Merck, 99.95 %) and ultrasonicated in a water bath at room temperature for t = 15 min. Subsequently, 7 µL of the dispersion were transferred to the topside of an Au-supported lacey carbon grid placed on top of a standard laboratory filter paper. This procedure was repeated twice. Prior to TEM measurements and after establishing a base pressure p_{base} = 0.08 mbar, the Au lacey carbon grids loaded with the specimen were treated for 45 s in a He plasma (p_{He} = 0.3 mbar, power: P = 20 W, f = 40 kHz; Zepto CE 40 kHz, Diener electronic – Plasma-Surface-Technology).

1.4. Elemental Analysis

For a quantitative analysis of the elemental composition, the element specific probabilities of X-ray emission have been taken into account by applying the Cliff-Lorimer method. This method is used for thin samples (~ 10 nm), where additional factors influencing the yield of X-ray emission such as the absorption of emitted characteristic X-rays by the specimen or fluorescence originating from atoms in an excited state leading to emission of secondary characteristic X-rays are negligible. The concentration ratio of the elements is proportional (proportionality factor k_{CL}) to the intensity ratio of the characteristic X-rays emitted from the elements. The factor k_{CL} accounts specifically for the cross section for the generation of characteristic X-rays from the element of interest and the reference element.[4] The intensities of the characteristic X-rays were obtained by integrating over the counts originating from element specific transitions. Mn was quantified considering the absorption edge of the Mn Kα transition (X-ray energy: E = 5.894 keV) and integrating counts between 5.740 keV and 6.050 keV and the Zn content was obtained by integrating the counts originating from the Zn Kα transition between 8.440 keV and 8.820 keV (X-ray energy: E = 8.630 keV).

2. Supplementary Data

2.1. Additional Figures

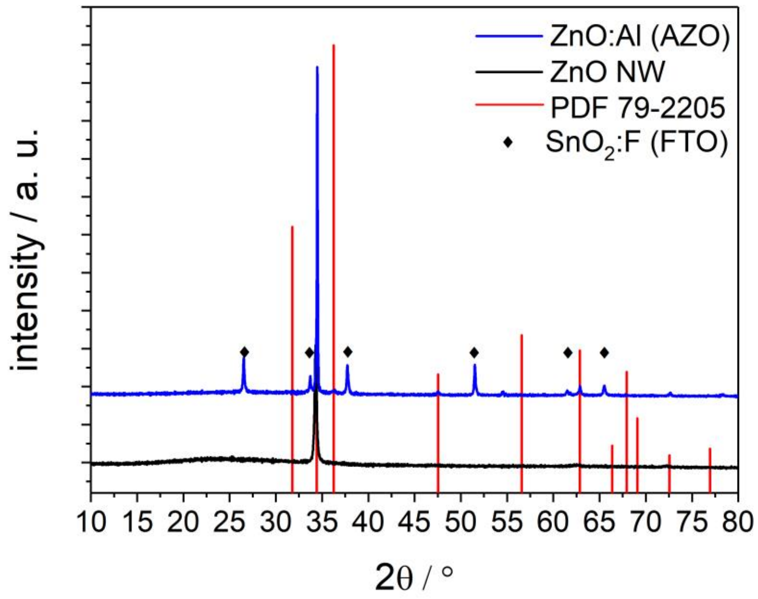

Figure S1: X-ray diffraction patterns of an AZO (aluminum-doped zinc oxide, ZnO:Al) film on a FTO (fluorine-doped tin oxide, SnO_2:F) covered glass substrate before (blue line) and after (black line) electrodeposition of a ZnO nanowire (ZnO NW) film. Reference data for the ZnO wurtzite phase correspond to PDF 79-2205. Diamonds represent reflexes originating from cassiterite (PDF 41-1445).

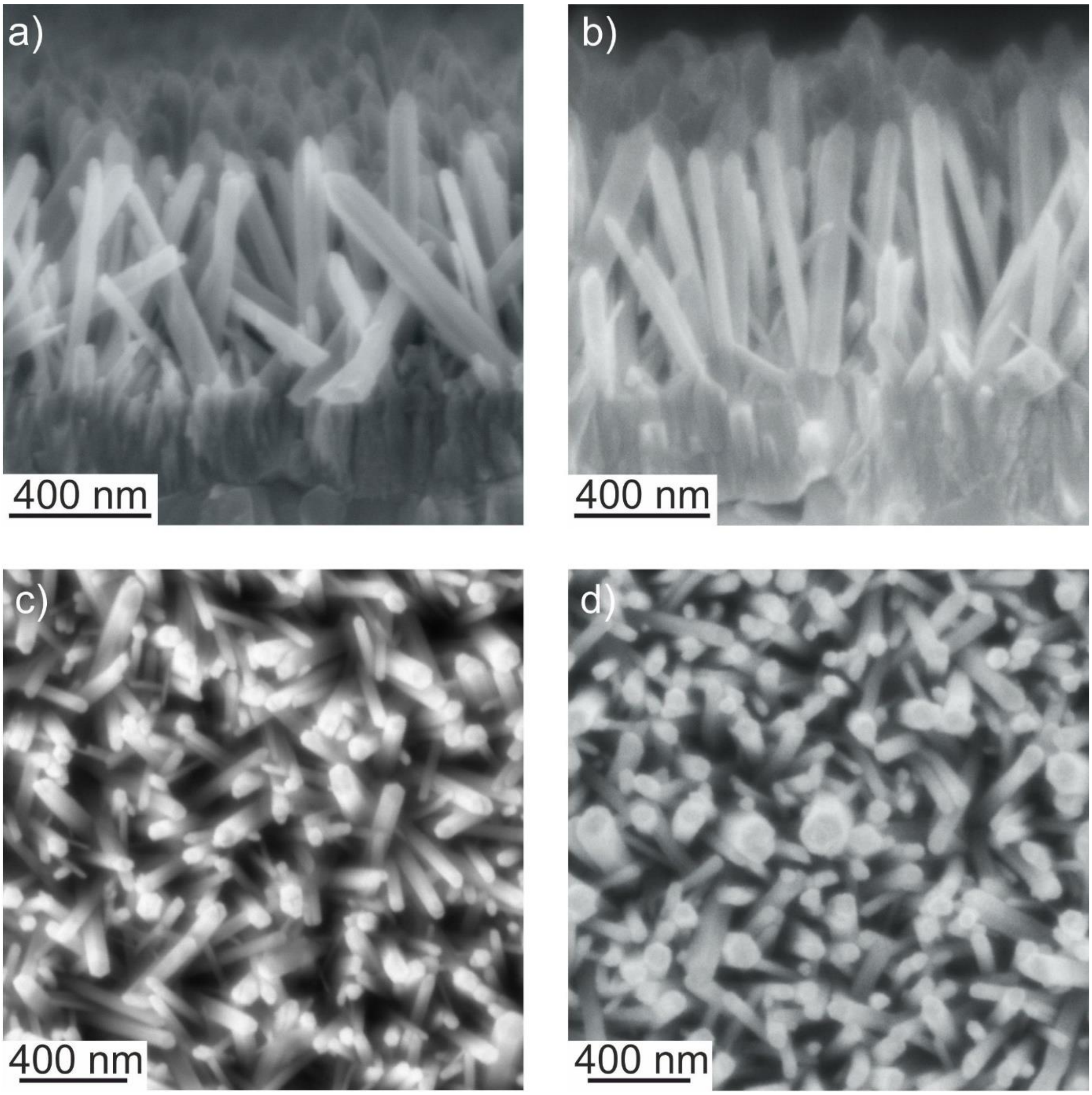

Figure S2: Scanning electron micrographs of the cross sections (a,b) and of top views (c,d) of ZnO nanowire electrodes (a,c) before and (b,d) after electrodeposition for 120 min at $E_{Ag/AgCl}$ = 0.376 V in a N_2-purged 0.175 mM $KMnO_4$ aqueous solution.

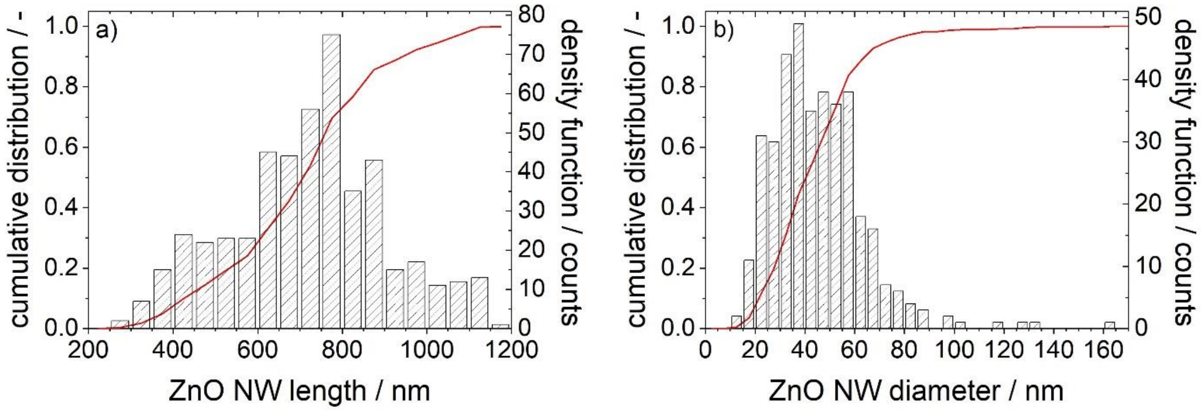

Figure S3: Distribution of the length (a) and the diameter (b) of ZnO nanowires (ZnO NW) as determined from scanning electron micrographs of electrode cross sections.

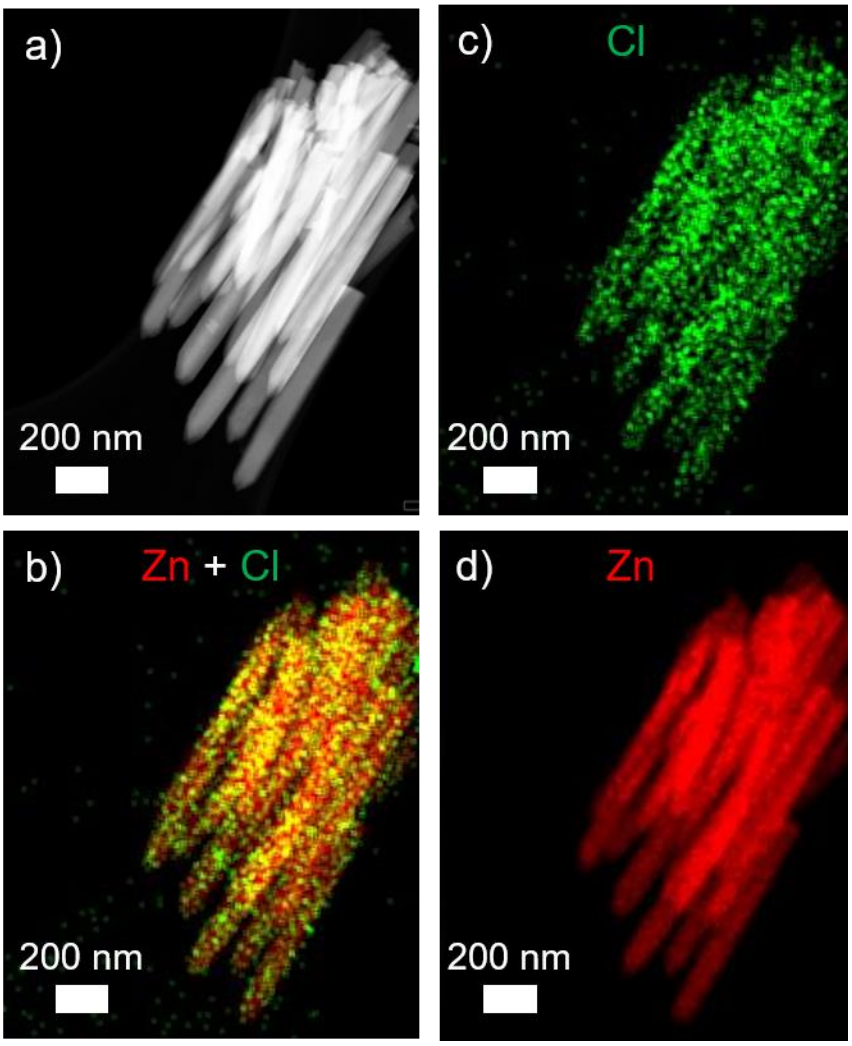

Figure S4: STEM-HAADF image (a) and elemental intensity maps (b-d) as obtained by EDX analysis of ZnO nanowires. Single elemental maps of Cl (c) and Zn (d) are combined to a mixed elemental map (b).

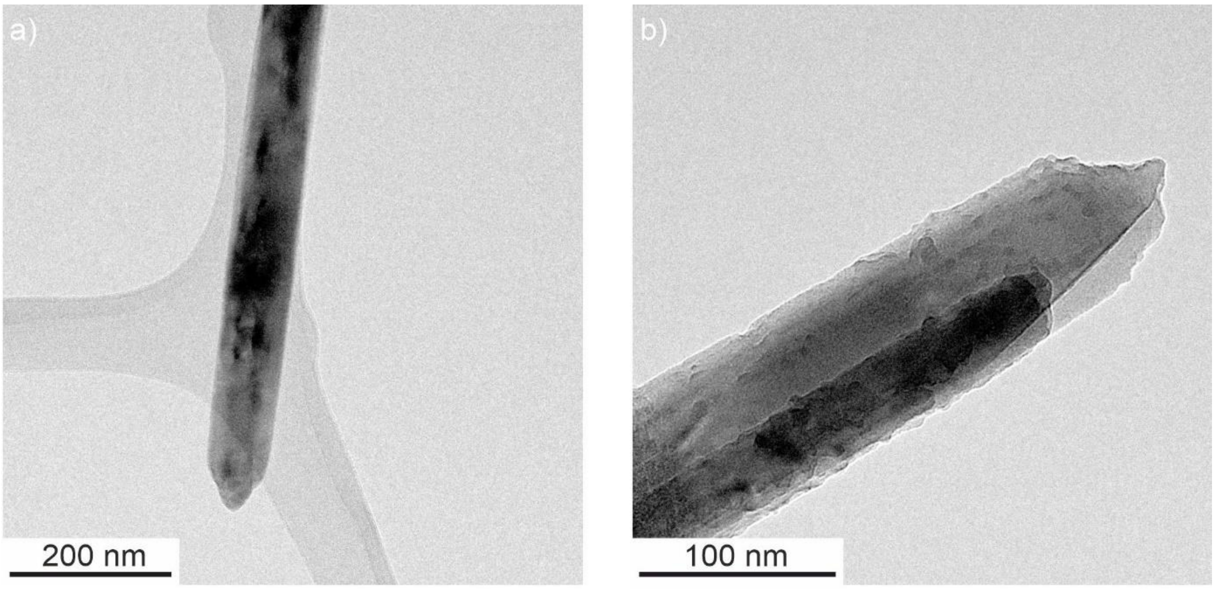

Figure S5: Transmission electron micrographs of representative single nanowires from a ZnO nanowire array (a) before and (b) after electrodeposition for 45 min at $E_{Ag/AgCl}$ = 0.376 V in a N_2-purged 0.175 mM $KMnO_4$ aqueous solution.

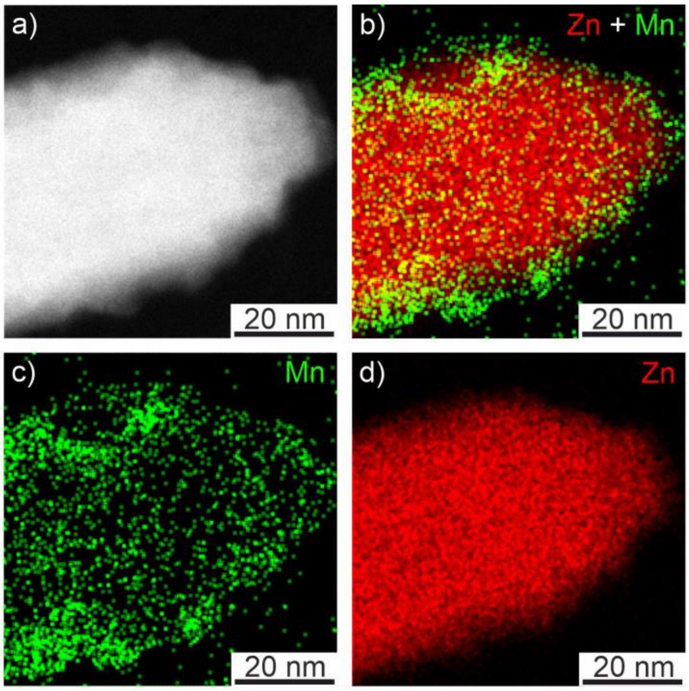

Figure S6: STEM-HAADF image (a) and elemental intensity maps (b-d) as obtained by EDX analysis of composite nanostructures resulting from electrodeposition at $E_{Ag/AgCl}$ = 0.376 V in aqueous $KMnO_4$ solution (deposition time: 45 min). Single elemental maps of Mn (c) and Zn (d) are combined to a mixed elemental map (b).

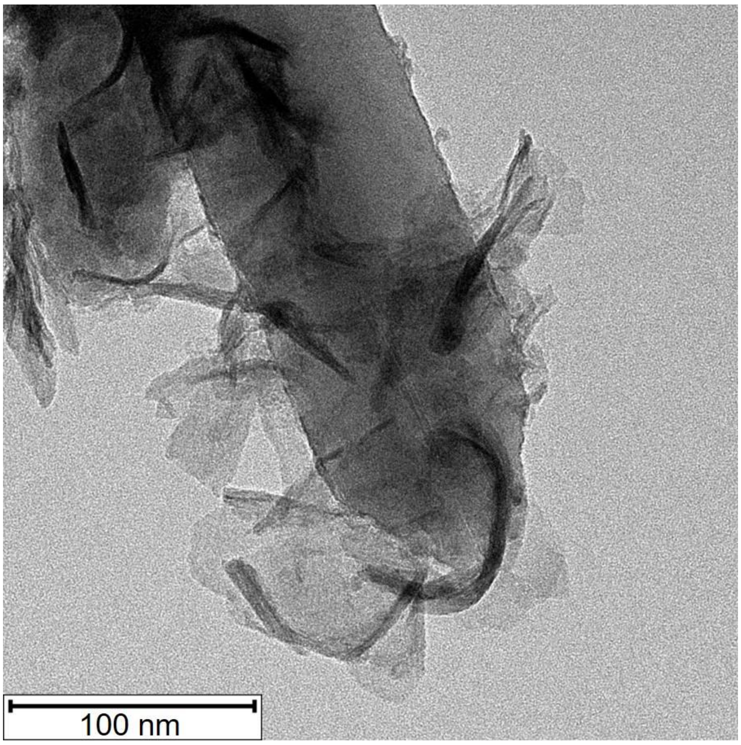

Figure S7: High resolution TEM image of the top region of ZnO nanowires following electrodeposition at $E_{Ag/AgCl}$ = 0.000 V in aqueous $KMnO_4$ solution (deposition time: 45 min).

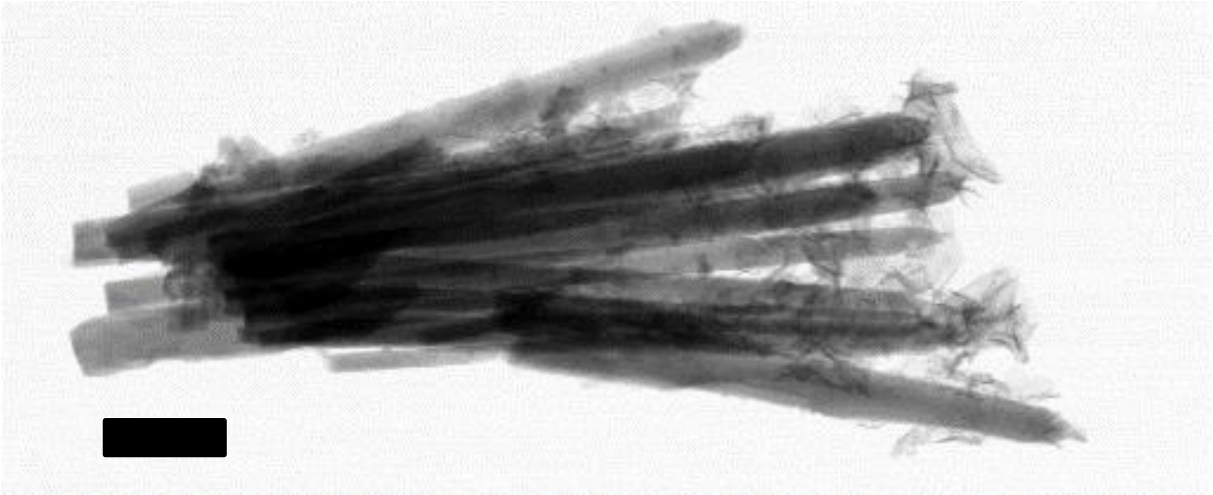

Figure S8: STEM-bright field image of ZnO nanowires after electrodeposition at $E_{Ag/AgCl}$ = 0.000 V in aqueous $KMnO_4$ solution (deposition time: 45 min). Scale bar: 200 nm.

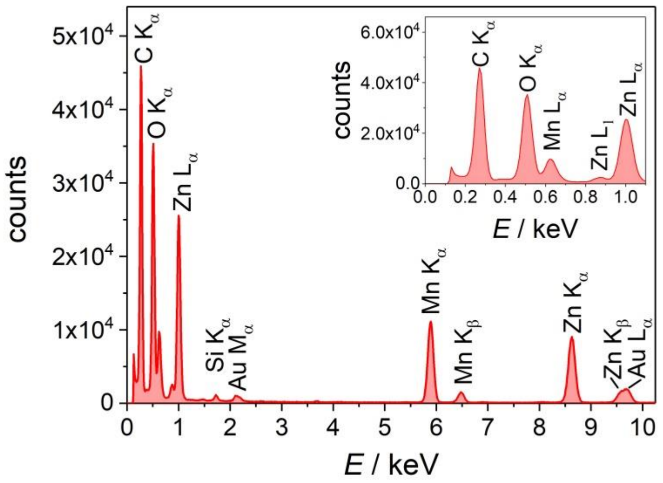

Figure S9: Representative EDX spectrum of the electrodeposit, which was grown on ZnO nanowires at $E_{Ag/AgCl}$ = 0.000 V in aqueous $KMnO_4$ solution.

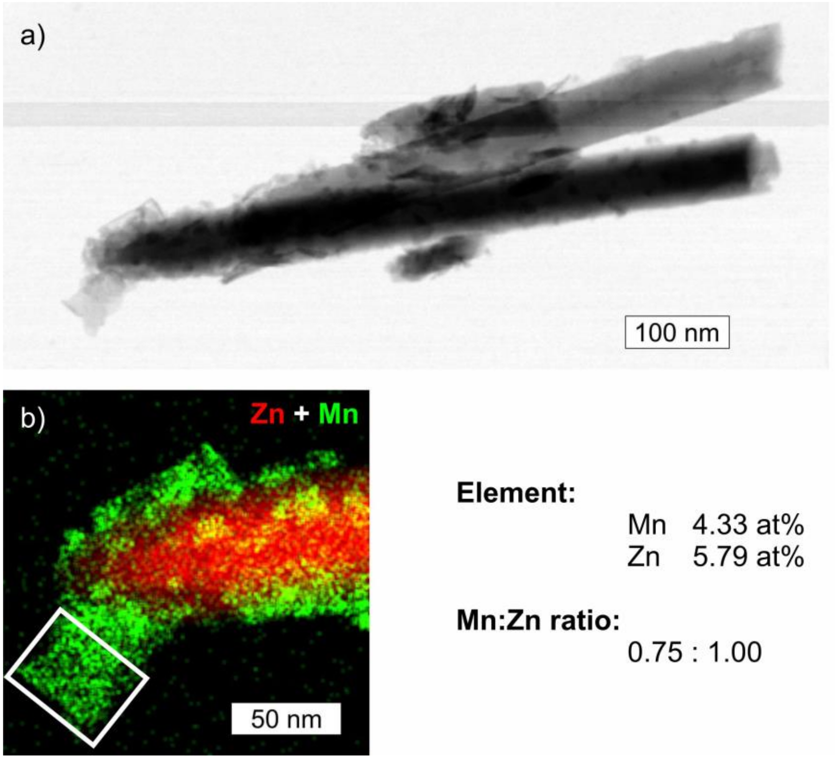

Figure S10: (a) STEM bright field image and (b) mixed (Zn + Mn) elemental map as obtained by EDX analysis of composite nanostructures resulting from electrodeposition at $E_{Ag/AgCl}$ = 0.000 V in aqueous $KMnO_4$ solution (deposition time: 45 min). For quantification of the elemental composition, the element specific probabilities of X-ray emission have been taken into account by applying the Cliff-Lorimer method. For this purpose, the sample region indicated by the white square in the mixed elemental map (b) was analysed.

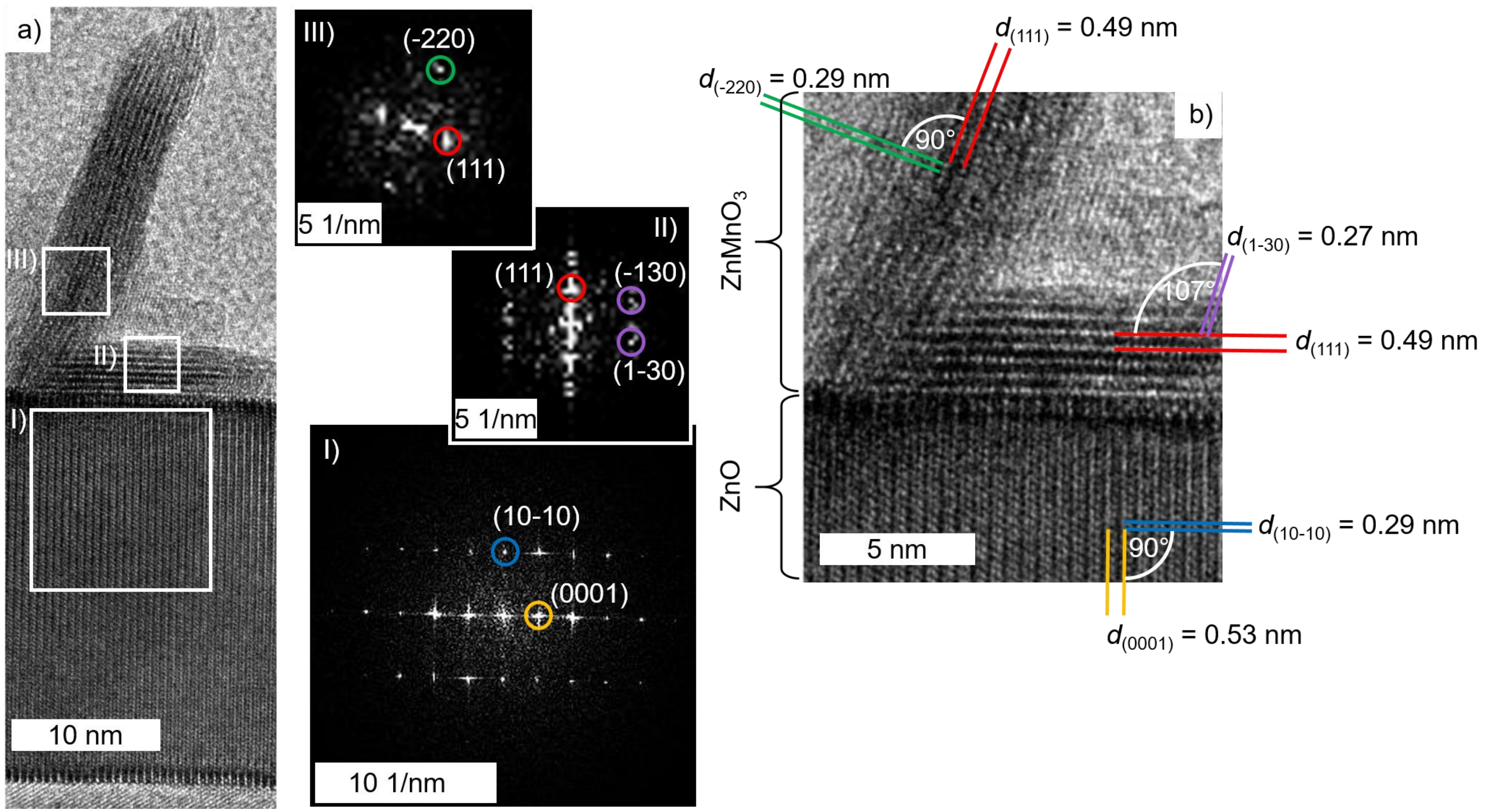

Figure S11: (a) High resolution TEM image of a $ZnMnO_3$/ZnO nanocomposite. White squares indicate regions (I-III), which were selected for FFT analysis. (I-III) FFT patterns of sample spots indicated by squares in (a). Assignment of FFT spots to lattice planes is based on the analysis of lattice spacings and relative orientation of lattice planes in hexagonal wurtzite ZnO and defective cubic spinel $ZnMnO_3$. b) Magnification of the sample region featuring the solid/solid interface. Identified lattice planes are indicated together with the respective interplanar spacings.

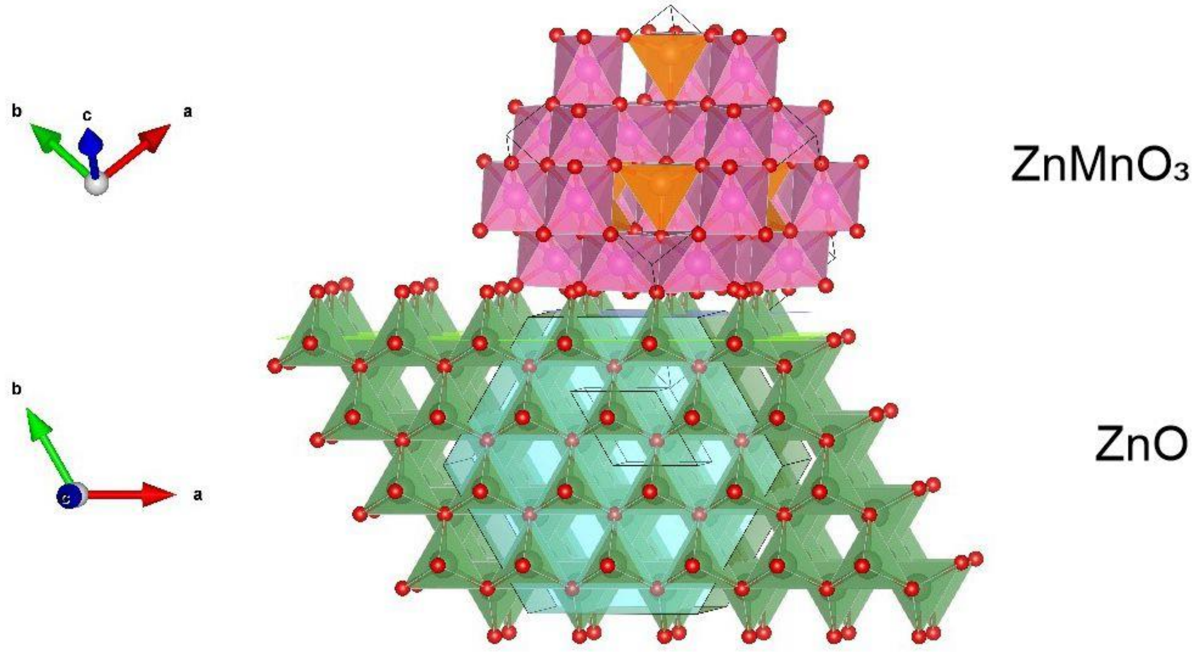

Figure S12: Scheme of the interface formed between the (111) plane of cubic spinel $ZnMnO_3$ and the hexagonal wurtzite ZnO ($10\bar{1}0$) plane. Clusters were separately constructed using the VESTA software[5] and then merged by hand to obtain a scheme of the composite structure. Magenta octahedra correspond to [MnO_6] units, orange ($ZnMnO_3$) and green (ZnO) tertahedra represent [ZnO_4] units. Lattice parameters of cubic spinel $ZnMnO_3$ and of hexagonal wurtzite ZnO were taken from references [6] and [7], respectively.

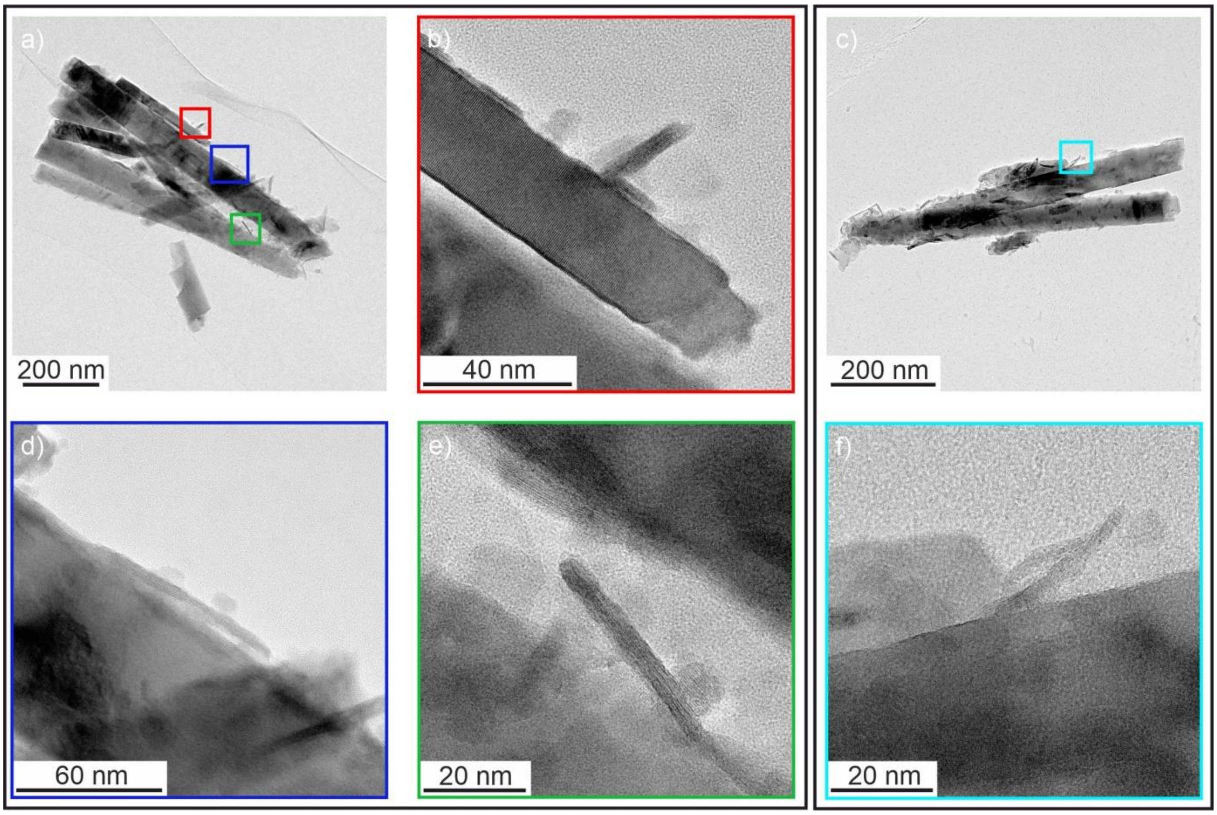

Figure S13: (a,c) Transmission electron micrographs and (b,d-f) high resolution TEM images (corresponding to sample regions indicated by squares in (a) and (c), respectively) of ZnO nanowires after electrodeposition at $E_{Ag/AgCl}$ = 0.000 V in aqueous $KMnO_4$ solution (deposition time: 45 min).

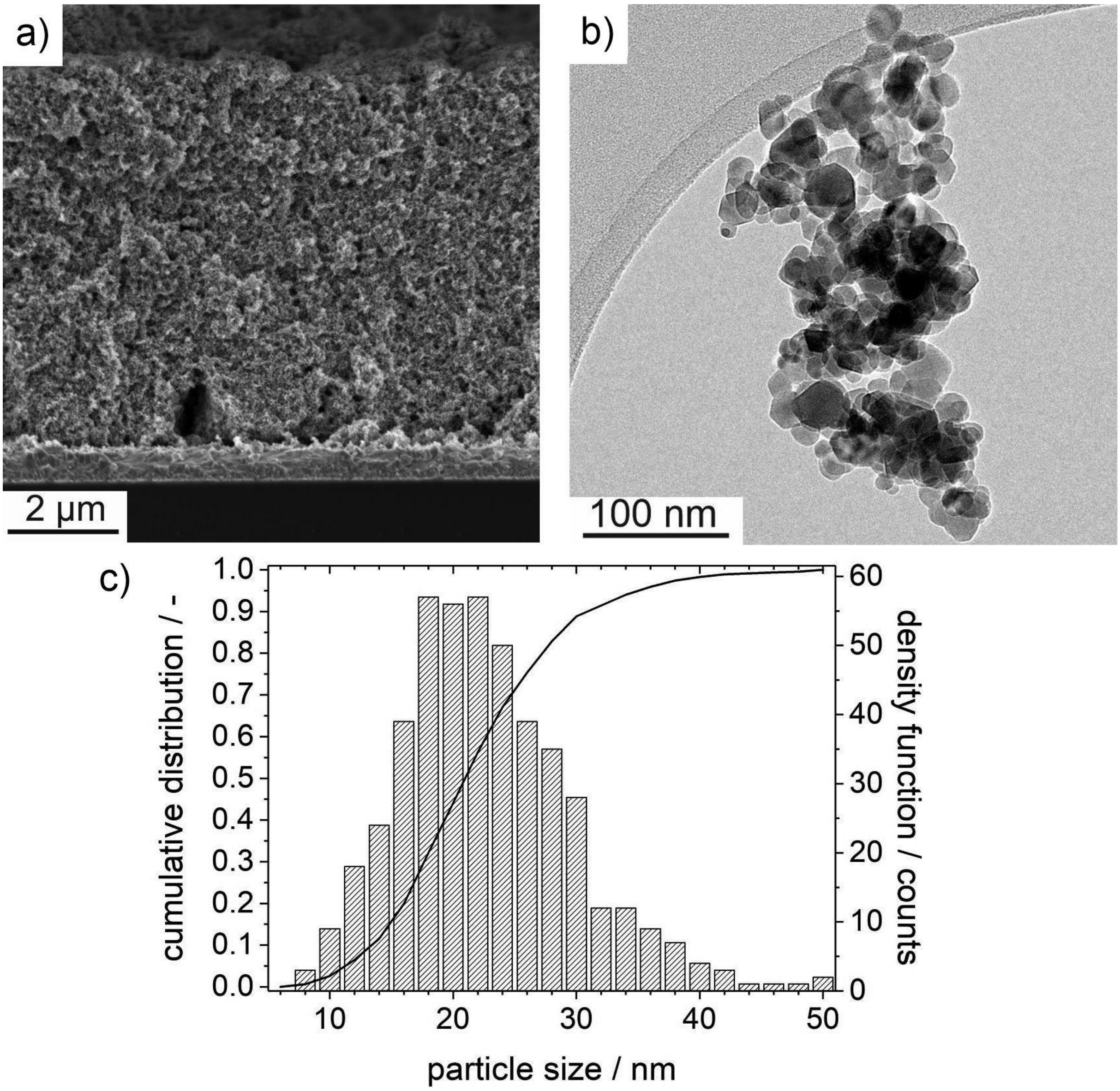

Figure S14: (a) Scanning electron micrograph of the cross section of a ZnO nanoparticle film sintered at 450 °C for 1 h. (b) Transmission electron micrograph of ZnO nanoparticle aggregates. TEM samples were prepared by scratching a part of the ZnO nanoparticle film from the glass substrate and immobilization on a lacey carbon grid. (c) Particle size distribution of primary particles.

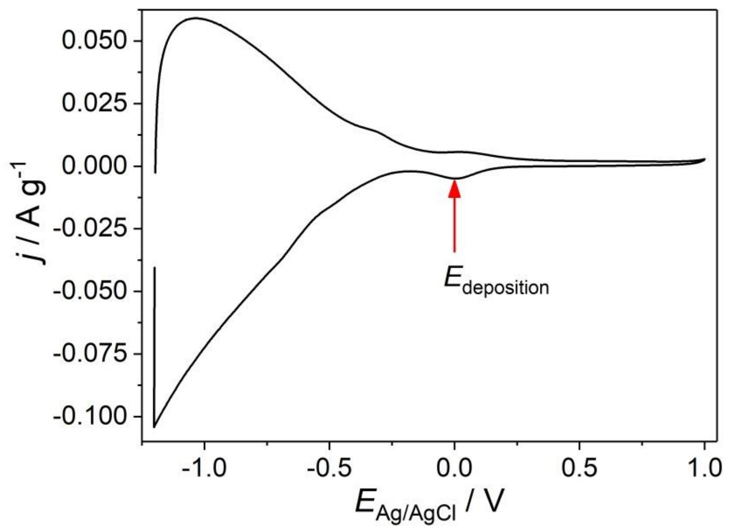

Figure S15: Cyclic voltammogram of a ZnO nanoparticle electrode. The red arrow marks the potential, at which $ZnMnO_3$ electrodeposition was performed. Electrolyte: 1.0 M Na_2SO_4 purged with N_2.

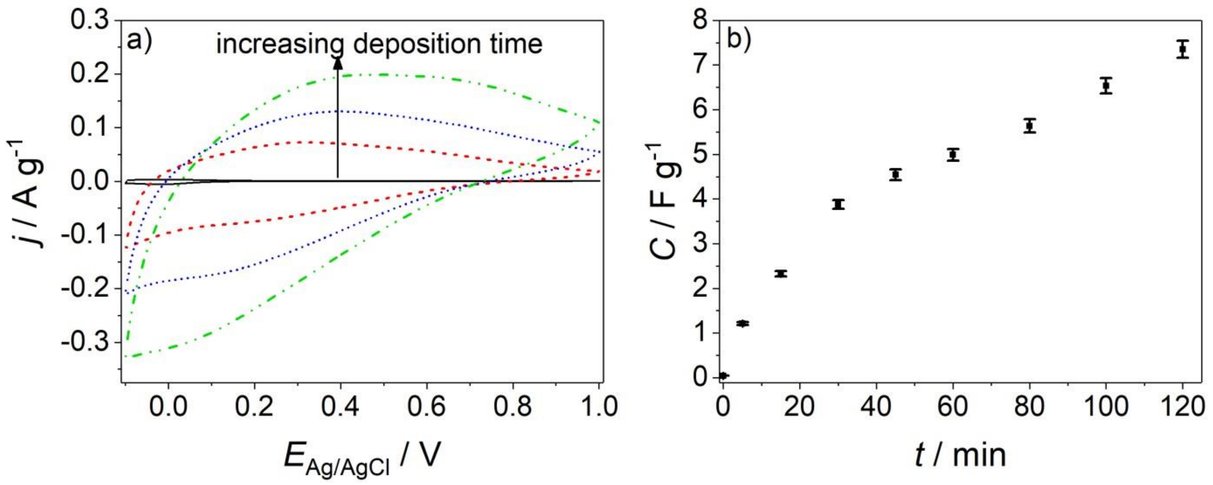

Figure S16: (a) Cyclic voltammograms recorded at a scan rate v = 0.020 V s^{-1} in nitrogen-purged 1 M Na_2SO_4 aqueous solution for ZnO nanoparticle electrodes before (black, solid lines) and after sequential deposition of $ZnMnO_3$ at an electrodeposition potential of $E_{Ag/AgCl}$ = 0.000 V. Electrodeposition time: 15 min (red, dashed lines), 45 min (blue, dotted lines), and 120 min (green, dashed and dotted lines); electrodeposition solution: N_2-purged 0.175 mM $KMnO_4$ aqueous solution. After each electrodeposition step electrodes were washed with ultrapure water before recording voltammograms in 1 M Na_2SO_4 solution. (b) Specific capacitance (as calculated from data in (a)) as a function of deposition time. The specific capacitance is referenced to the total electrode mass (i.e. the mass of ZnO nanoparticles and of the $ZnMnO_3$ deposit).

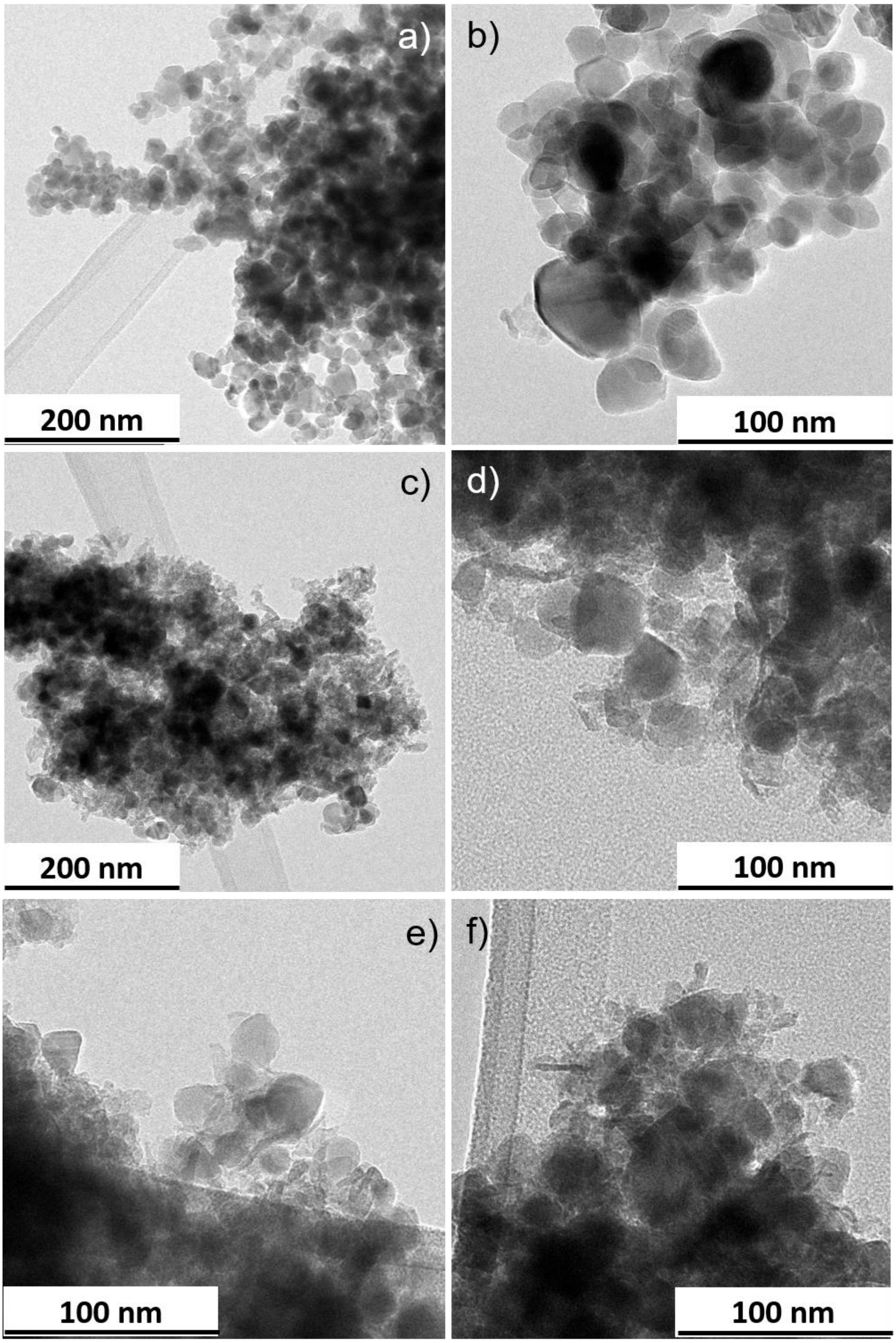

Figure S17: Transmission electron micrographs of nanoparticle aggregates as removed from a ZnO nanoparticle film after electrodeposition at $E_{Ag/AgCl}$ = 0.000 V in a N_2-purged 0.175 mM $KMnO_4$ aqueous solution for 120 min. The sample was scratched from the glass substrate and immobilized on a lacey carbon grid for TEM measurements.

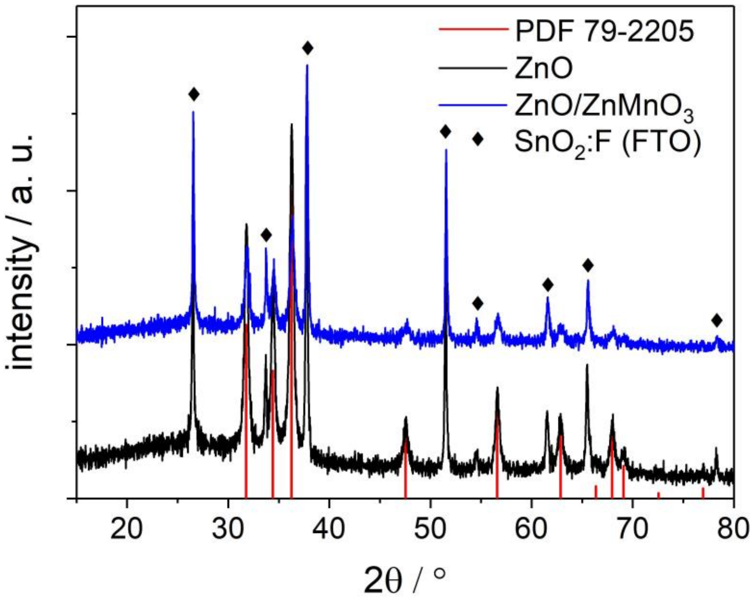

Figure S18: X-ray diffraction patterns of a ZnO nanoparticle film on a FTO (fluorine-doped tin oxide, SnO_2:F) covered glass substrate before (black line) and after (blue line) electrodeposition of $ZnMnO_3$ from an 0.175 mM $KMnO_4$ aqueous solution (t = 45 min, $E_{Ag/AgCl}$ = 0.000 V). Reference data for the ZnO wurtzite phase correspond to PDF 79-2205. Diamonds represent reflexes originating from cassiterite (PDF 41-1445)

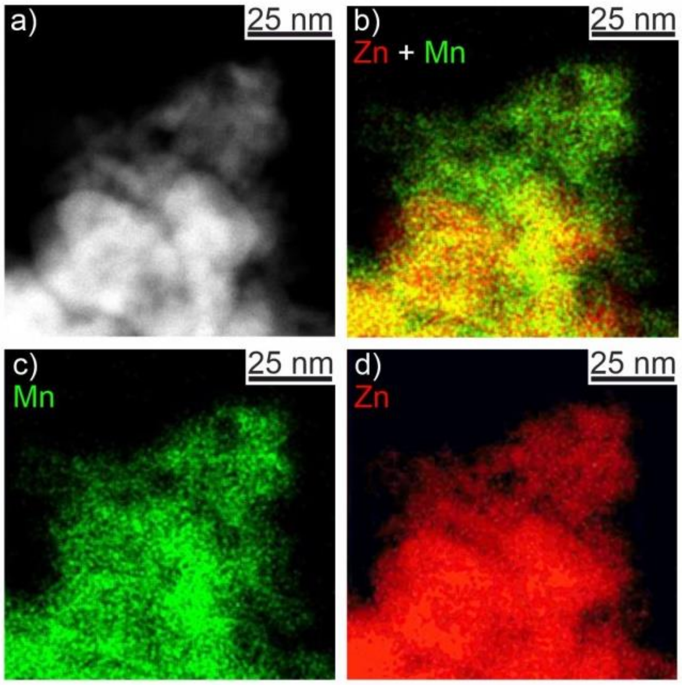

Figure S19: STEM-HAADF image (a) and elemental intensity maps (b-d) as obtained by EDX analysis of ZnO/$ZnMnO_3$ composites based on ZnO nanoparticle films and resulting from electrodeposition at $E_{Ag/AgCl}$ = 0.000 V in aqueous $KMnO_4$ solution (deposition time: 120 min). Single elemental maps of Mn (c) and Zn (d) are combined to a mixed elemental map (b).

2.2. Additional Tables

Table S1: Comparison of experimentally determined values of lattice spacings in defective cubic spinel $ZnMnO_3$ (Figure 6) with literature values.[6]

phase	lattice plane	d_{ref} / Å [Ref. [6]]	d_{exp} / Å		
			Figure 6 (I)	Figure 6 (II)	Figure 6 (III)
$ZnMnO_3$	(111)	4.8179	-	4.9 ± 0.1	4.9 ± 0.1
	(310)/(311)	2.6388/2.5161	2.7 ± 0.1	-	2.5 ± 0.1
	(400)	2.0862	-	-	2.1 ± 0.1

Table S2: Comparison of experimentally determined values of lattice spacings and relative crystallographic orientation of lattice planes in hexagonal wurtzite ZnO and defective cubic spinel $ZnMnO_3$ (Figure S11) with literature values.

phase	lattice plane	d_{ref} / Å	d_{exp} / Å	$angle_{calc}$ / °	$angle_{exp}$ / °
$ZnMnO_3$	(111)	4.8179 [Ref. [6]]	4.9 ± 0.1	-	-
$ZnMnO_3$	(-220)	2.9503 [Ref. [6]]	2.9 ± 0.1	$q_{(111)/(-220)}$: 90	$q_{(111)/(-220)}$: 90 ± 3
$ZnMnO_3$	(1-30)	2.6388 [Ref. [6]]	2.7 ± 0.1	$q_{(111)/(1-30)}$: 111	$q_{(111)/(1-30)}$: 107 ± 3
ZnO	(0001)	5.2065 [Ref. [7]]	5.3 ± 0.1	-	-
ZnO	(10-10)	2.8544 [a]	2.9 ± 0.1	$q_{(0001)/(10-10)}$: 90	$q_{(0001)/(10-10)}$: 90 ± 1

[a] Calculated value[8] using the lattice parameters of hexagonal ZnO: a = 3.296 Å and c = 5.2065 Å.[7]

References

1 J. Elias, R. Tena-Zaera and C. Lévy-Clément, Electrodeposition of ZnO nanowires with controlled dimensions for photovoltaic applications: Role of buffer layer, *Thin Solid Films*, 2007, **515**, 8553–8557.

2 A. R. Gheisi, C. Neygandhi, A. K. Sternig, E. Carrasco, H. Marbach, D. Thomele and O. Diwald, O_2 adsorption dependent photoluminescence emission from metal oxide nanoparticles, *Physical Chemistry Chemical Physics*, 2014, **16**, 23922–23929.

3 K. Kocsis, M. Niedermaier, J. Bernardi, T. Berger and O. Diwald, Changing interfaces: Photoluminescent ZnO nanoparticle powders in different aqueous environments, *Surface Science*, 2016, **652**, 253–260.

4 C. B. Carter and D. B. Williams, *Transmission Electron Microscopy: Diffraction, Imaging, and Spectrometry*, Springer International Publishing, 2016.

5 K. Momma and F. Izumi, VESTA3 for three-dimensional visualization of crystal, volumetric and morphology data, *Journal of Applied Crystallography*, 2011, **44**, 1272–1276.

6 L. V. Saraf, P. Nachimuthu, M. H. Engelhard and D. R. Baer, Stabilization of $ZnMnO_3$ phase from sol-gel synthesized nitrate precursors, *Journal of Sol-Gel Science and Technology*, 2010, **53**, 141–147.

7 Z. L. Wang, Zinc oxide nanostructures: Growth, properties and applications, *Journal of Physics Condensed Matter*, 2004, **16**, R829-R858.

8 X. Zou, S. Hovmöller and P. Oleynikov, *Electron Crystallography: Electron Microscopy and Electron Diffraction*, 2012.
